# VEGF receptor heterodimers and homodimers are differentially expressed in neuronal and endothelial cell types

**DOI:** 10.1371/journal.pone.0269818

**Published:** 2022-07-21

**Authors:** Joy Sarkar, Yuncin Luo, Qiang Zhou, Evguenia Ivakhnitskaia, Daniel Lara, Eitan Katz, Michael G. Sun, Victor Guaiquil, Mark Rosenblatt

**Affiliations:** Department of Ophthalmology and Visual Sciences, Illinois Eye and Ear Infirmary, University of Illinois at Chicago, College of Medicine, Chicago, Illinois, United States of America; Huntington Medical Research Institutes, UNITED STATES

## Abstract

**Purpose:**

We have previously reported that VEGF-B is more potent than VEGF-A in mediating corneal nerve growth *in vitro* and *in vivo*, and this stimulation of nerve growth appears to be different from stimulation of angiogenesis by these same ligands, at least in part due to differences in VEGF receptor activation. VEGF signaling may be modulated by a number of factors including receptor number or the formation of receptor hetero- vs. homodimers. In endothelial cells, VEGF receptor heterodimer (VEGR1/R2) activation after ligand binding and subsequent phosphorylation alters the activation of downstream signaling cascades. However, our understanding of these processes in neuronal cell types remains unclear. The purpose of this study was to identify the presence and distribution of VEGF Receptor-Ligand interactions in neuronal cells as compared to endothelial cells.

**Methods:**

PC12 (rat neuronal cell line), MAEC (mouse aortic endothelial cell line), MVEC (mouse venous endothelial cell line) and HUVEC (human umbilical venous endothelial cell line; control group) were used. Cells were acutely stimulated either with VEGF-A (50 ng/μL) or VEGF-B (50 ng/μL) or “vehicle” (PBS; control group). We also isolated mouse trigeminal ganglion cells from *thy1*-YFP neurofluorescent mice. After treatment, cells were used as follows: (i) One group was fixed in 4% paraformaldehyde and processed for VEGFR1 and VEGFR2 immunostaining and visualized using confocal fluorescence microscopy and Total Internal Reflection (TIRF) microscopy; (ii) the second group was harvested in cell lysis buffer (containing anti-protease / anti-phosphatase cocktail), lysed and processed for immunoprecipitation (IP; Thermo Fisher IP kit) and immunoblotting (IB; LI-COR® Systems). Immunoprecipitated proteins were probed either with anti-VEGFR1 or anti-VEGFR2 IgG antibodies to evaluate VEGFR1-R2-heterodimerization; (iii) a third group of cells was also processed for Duolink Proximity Ligation Assay (PLA; Sigma) to assess the presence and distribution of VEGF-receptor homo- and heterodimers in neuronal and endothelial cells.

**Results:**

TIRF and fluorescence confocal microscopy revealed the presence of VEGFR1 co-localized with VEGFR2 in endothelial and PC12 neuronal cells. Cell lysates immunoprecipitated with anti-VEGFR1 further validated the existence of VEGFR1-R2 heterodimers in PC12 neuronal cells. Neuronal cells showed higher levels of VEGFR1-R2 heterodimers as compared to endothelial cells whereas endothelial cells showed higher VEGFR2-R2 homodimers compared to neuronal cells as demonstrated by Duolink PLA. Levels of VEGFR1-R1 homodimers were very low in neuronal and endothelial cells.

**Conclusions:**

Differences in VEGF Receptor homo- and heterodimer distribution may explain the differential role of VEGF ligands in neuronal versus endothelial cell types. This may in turn influence VEGF activity and regulation of neuronal cell homeostasis.

## Introduction

VEGF (Vascular Endothelial Growth Factor) remains the most well-studied pro-angiogenic molecule belonging to a family of homodimeric growth factors which signal through receptor tyrosine kinases. First identified as a vascular permeability factor, VEGF is now recognized as a potent mitogen for endothelial cells and promoter of endothelial migration. More recently, pleiotropic roles for VEGF in wound healing, lymphangiogenesis and neuroprotection have been recognized [1–3]. Nerves and arteries share ligand and receptor pairs, and in many systems there is a congruence of nerve and vessel patterning. In some models, nerves appear to pattern vessels, in others the vessels pattern the nerves [4]. VEGF is now recognized as an important regulator of neural cell growth and function. VEGF and its receptors are expressed in the developing neural system and appear to regulate the development of key portions of the CNS. VEGF exhibits both neurotrophic and neuroprotective effects for CNS and PNS neurons in culture [5–8]. Dorsal root ganglion cells of the PNS had more axonal growth and more axonal branching when stimulated with VEGF [9, 10]. The actions of VEGF on neurons appear to be mediated in most cases through VEGFR-2 signaling, but some isolated reports of VEGFR-1 signaling have been reported. While VEGF signaling has been well studied in the field of angiogenesis, there is a paucity of information regarding the mechanism by which VEGF can induce nerve regeneration. Prior studies from our lab [5, 11, 12] have shed some light on the fact that there are fundamental differences in angiogenesis and nerve signaling highlighted by the finding that the relatively non-angiogenic ligand VEGF-B has potent roles in the peripheral nervous system (PNS). These unpredicted potencies of VEGF-B may be due to differences in VEGFR1 homodimer presence, activation or signaling in neuronal cell types, or perhaps due to previously undiscovered VEGFR1:VEGR2 heterodimers in neuronal cells.

In our current study, we investigated the existence of VEGFR1 and R2 homodimers and heterodimers in neuronal cells and characterized similarities and differences in their expression levels and distribution as compared to endothelial cells.

## Materials and methods

### Animals

All animal experiments were conducted in strict accordance with the recommendations in the Guide for the Care and Use of Laboratory Animals of the National Institutes of Health as well as the guidelines of the Association for Research in Vision and Ophthalmology Statement for the Use of Animals in Ophthalmic and Vision Research. The animal protocol was approved by the Institutional Animal Care and Use Committee (IACUC) of the University of Illinois at Chicago (Protocol Number: 20–222). *Thy1*-YFP neurofluorescent homozygous adult mice (6–8 weeks old) were purchased from Jackson Laboratories (Bar Harbor, ME), and colonies were established by inbreeding. For *ex vivo* experiments, mice were anesthetized with intraperitoneal injections of ketamine (20 mg/kg; Phoenix Scientific, St. Joseph, MO) and xylazine (6 mg/kg; Phoenix Scientific). For terminal experiments, mice were sacrificed by CO2 inhalation followed by cervical dislocation according to the IACUC protocol. All efforts were made to minimize suffering.

### Cell culture

PC12 rat pheochromocytoma cells were originally obtained from the American Type Culture Collection (CRL-1721; ATCC, Manassas, VA). MAEC (mouse aortic endothelial cell line), MVEC (mouse venous endothelial cell line) and HUVEC (human umbilical venous endothelial cell line; used as an “endothelial cell control”) in our studies were gifts from Dr. Dimitri Azar of University of Illinois Chicago [13]. Induced PC12 cells were grown on 100mm tissue culture petridishes pre-coated with collagen substrate, Collagen I solution (0.5 mg/mL, BD Biosciences) and maintained in RPMI-1640 medium supplemented with 10% heat-inactivated horse serum and 5% fetal bovine serum (FBS), 100 U/ml penicillin G, and 100 μg/ml streptomycin (Gibco, Grand Island, NY) and 50 ng/ml purified recombinant Mouse beta-NGF (R&D Systems) at 37°C under an atmosphere of 5% CO2 and 95% air. HUVEC, MAEC and MVEC were maintained in VascuLife® Endothelial Medium (containing EnGS (containing Endothelial Cell Growth Supplement; Lifeline® Cell Technology, Frederick, MD) at 37°C under an atmosphere of 5% CO2 and 95% air.

### Trigeminal neuronal cell culture

Trigeminal ganglia (TG) were isolated from *thy1*-YFP (yellow fluorescent protein) transgenic mice in which the nerves show yellow fluorescence and cultured as described previously [12, 14]. In brief, ophthalmic branches of the trigeminal nerves were harvested and cleaned from contaminating tissue, then subjected to enzymatic digestion with papain and collagenase/dispase (Worthington Biochemicals, Lakewood, NJ) and separated in Percoll gradients by centrifugation. Cell cultures were maintained in 35 mm glass-bottom dishes coated with 20 μg/mL laminin/poly-D-Lysine (Sigma, St. Louis, MO) and supported with media (Neurobasal A medium supplemented with 1% B27; Invitrogen, Carlsbad, CA) supplemented with 2% fetal bovine serum (FBS) and 1% antibiotic/antimycotic (ABAM; Gibco, Grand Island, NY).

### Immunofluorescence microscopy

Cells were stimulated with either VEGF-A (50 ng/ml; R&D Systems, Minneapolis, MN) or VEGF-B (50 ng/ml; R&D Systems, Minneapolis, MN) for 15 min. Following stimulation, cells were fixed in 4% paraformaldehyde for 15 min. Fixed cells were then washed 3 times in PBS, blocked (2% BSA, 2.5% donkey serum in PBS) for 1h at room temperature and incubated overnight with 1:200 dilution of rat anti-VEGFR1 (R&D Systems, Minneapolis, MN) or rabbit anti-VEGFR2 antibody (Proteintech, Rosemont, IL) at 4˚C. Cells were washed 3 times in PBS and incubated at room temperature for 1 hour with 1:400 dilution of AlexaFluor488-conjugated goat anti-rat IgG (Life Technologies, Carlsbad, CA) or donkey anti-rabbit Cy3 secondary antibody at 1:500 dilution (Jackson Immunoresearch Laboratories, West Grove, PA). Cells were washed 3 times with PBS and mounted in Vectashield mounting medium with DAPI (Vector labs, Burlingame, CA). Immunofluorescence analysis was performed using 40X and 63X objectives on a Zeiss 710 Confocal Microscope and Zen Imaging Software (version 2.1; Carl Zeiss, GmbH, Hamburg, Germany) located in the UIC-Ophthalmology Imaging Research Core.

### Western immunoblot analysis

Cells were stimulated with either VEGF-A (50 ng/ml; R&D Systems, Minneapolis, MN; VEGFR1 and VEGFR2 binding) or VEGF-B (50 ng/ml; R&D Systems, Minneapolis, MN; ostensible VEGFR1 binding) for 15 min. Following stimulation, cells were lysed in a modified RIPA cell lysis buffer (20 mM Tris·HCl, pH 7.4, 150 mM NaCl, 1 mM EDTA, 1 mM EGTA, 1% IGEPAL, 2.5 mM sodium pyrophosphate, 1 mM β-glycerophosphate, pH 7.4) supplemented with a complete protease inhibitor and a phosphatase inhibitor Cocktail I and II (Sigma Chemical Co., St. Louis, MO). Samples were then centrifuged at 10,000g for 15 minutes at 4°C, and the supernatant (cell lysate) was collected. Total protein was determined using a modified Lowry method (BioRad DC Protein assay, BioRad Laboratories, Hercules, CA). For Western blot analysis, 50 μg total protein was electrophoretically run on 4% to 12% Tris–glycine SDS polyacrylamide gel (XCell SureLock Mini-Cell Electrophoresis System, Invitrogen). Samples were transferred to 0.2-μm nitrocellulose membranes (Whatman Inc., Florham Park, NJ) by electro-elution. Membranes were blocked in Li-Cor blocking buffer (Li-Cor Biosciences, Lincoln, NE), followed by incubation overnight at 4°C with either rat anti-VEGFR1 (1:500; R&D Systems, Minneapolis, MN) or rabbit anti-VEGFR2 antibody (1:500; Proteintech, Rosemont, IL) diluted in blocking buffer. Rabbit polyclonal anti-GAPDH antibody (1:2000; Cell Signaling, Danvers, MA) was used as a loading control. After three 10-minute washes in PBS containing 0.1% Tween-20, the blots were incubated for 2 hours at room temperature in the fluorescently labeled secondary antibody mixture (Rockland Immunoresearch, Gilbertsville, PA) of goat anti-mouse (IRDye 800CW, 1:15,000) and goat anti-rabbit (IRDye700DX, 1:10,000) antibodies diluted in blocking buffer. Membranes were then imaged using LiCor Odyssey Infrared imager (Li-Cor Biosciences). The relative intensity of each band was determined with the Fiji NIH Image J software. Quantification was performed by subtracting background readings from the relative intensity for each sample band and normalizing it with that of GAPDH.

### Immunoprecipitation (IP)

For IP experiments, PC12, MAEC, MVEC and HUVEC (positive control) were lysed in a modified RIPA cell lysis buffer (20 mM Tris·HCl, pH 7.4, 150 mM NaCl, 1 mM EDTA, 1 mM EGTA, 1% IGEPAL, 2.5 mM sodium pyrophosphate, 1 mM β-glycerophosphate, pH 7.4) supplemented with a complete protease inhibitor and a phosphatase inhibitor Cocktail I and II (Sigma Chemical Co., St. Louis, MO). The cell lysates were immunoprecipitated using rat anti-VEGFR1 (R&D Systems, MN, USA) and DynaBeads® Protein G IP Kit (Thermo-Scientific, Invitrogen, CA, USA) as per manufacturer’s instructions. The Dynabeads®-Ab-Ag complexes obtained were denatured and processed for SDS–PAGE Western Immunoblotting and membranes probed with either rat anti-VEGFR1 (1:500; R&D Systems, Minneapolis, MN) or rabbit anti-VEGFR2 (1:500; Proteintech, Rosemont, IL). In order to evaluate tissue-specific differences in the distribution of VEGFR1-R2 heterodimers, tissue lysates such as mouse thoracic aorta, portal vein, dorsal root ganglia, trigeminal ganglia and cornea were also extracted and immunoprecipitated with rat anti-VEGFR1 antibody (R&D Systems, MN, USA) and DynaBeads® Protein G IP Kit (Thermo-Scientific, Invitrogen, CA, USA) as per manufacturer’s instructions. Bead only “negative” controls were used to account for non-specific VEGFR1 binding.

### Total internal reflection fluorescence (TIRF) microscopy

In order to visualize VEGFR1-R1 and VEGFR2-R2 homodimers and VEGFR1-R2 heterodimers, neuronal (PC12, TG neuronal) and endothelial (MAE, MVE) cells were fixed in 4% paraformaldehyde and probed with anti-VEGFR1 and anti-VEGFR2 antibodies and processed similar to the dual fluorescence staining protocol described earlier in the ‘Methods-Immunofluorescence Microscopy’ section. A Zeiss Total Internal Reflection Fluorescence (TIRF) microscope at the University of Illinois at Chicago Research Resources (UIC-RRC) Fluorescence Imaging Core was utilized for these studies.

### Proximal ligation assay (PLA) fluorescence immunostaining

PLA (Duolink®) allows the observation of protein-protein interactions within the cell, with clear, visual signals. The signal is generated only if the proteins of interest are located within 40nm, therefore detecting interaction. All procedures were performed as per manufacturer’s instructions (Millipore Sigma, USA). To estimate the PLA signals, the image data was analyzed for the mean fluorescence intensity of the PLA signals and/or the total number of PLA signals per cell or per area within the cell. Quantification was then reported as relative to technical and/or biological controls within a given experiment.

### Colocalization measurements

In fluorescence microscopy, colocalization refers to observation of the spatial overlap between two (or more) different fluorescent labels, each having a separate emission wavelength, to see if the different "targets" are located in the same area of the cell or very near to one another. For our experiments, using TIRF Microscopy, Fiji NIH Image J with the Colocalization threshold and Coloc2 plugin were used for colocalization analysis. The plugins used Pearson Correlation Coefficient (PCC). It is described as a measure of the linear correlation of intensity distribution between two variables and is not sensitive to differences in mean signal intensities or range, or image brightness or background or a zero offset between the two components. The values are +1 for perfect correlation, 0 for no correlation, and -1 for perfect anti-correlation. Based on published literature Table 1 depicts the rules for interpreting the size of a correlation coefficient.

**Table 1:** Interpreting the size of a correlation coefficient.

Size of Correlation	Interpretation
0.9–1.0	Very high positive correlation
0.7–0.9	High positive correlation
0.5–0.7	Moderate positive correlation
0.3–0.5	Low positive correlation
0–0.3	Negligible correlation

A correlation coefficient of 0–0.2 is considered to be negligible correlation, 0.2–0.3 is considered as low positive correlation, 0.5–0.7 is considered as moderate positive correlation, 0.7–0.9 is considered as high positive correlation and 0.9–1.0 is considered very high correlation [15, 16]. The Pearson Correlation Coefficient is regarded to be a superior measurement tool as compared to Mander’s Split Colocalization Coefficient based on published literature [17, 18].

### Statistical analyses

Statistical analyses were performed using GraphPad Prism Software. ANOVA and Student’s t-tests were used to compare mean values between groups. Results are shown as Mean ± SEM. Differences were considered significant if P ≤ 0.05.

## Results

While VEGF signaling has been well studied in the field of angiogenesis, there is a paucity of information regarding the mechanism of VEGF-induced nerve regeneration. The angiogenic effects of VEGF is predominantly dependent upon activation of VEGFR-2 (KDR, flk-1), but VEGF-A binds VEGFR-1 (flt-1) with high affinity as well. Upon VEGF binding, VEGFRs dimerize, undergoing cross-phosphorylation and activation of multiple cell signaling cascades [19–21]). Information on VEGF receptor expression in neurons is either lacking or unclear. Using microscopic imaging and biochemical techniques we characterized the expression of VEGFR1 and VEGFR2 in neuronal cells in comparison to endothelial cells.

### VEGFR1 and VEGFR2 visualization in neuronal and endothelial cells

**Fig 1:**
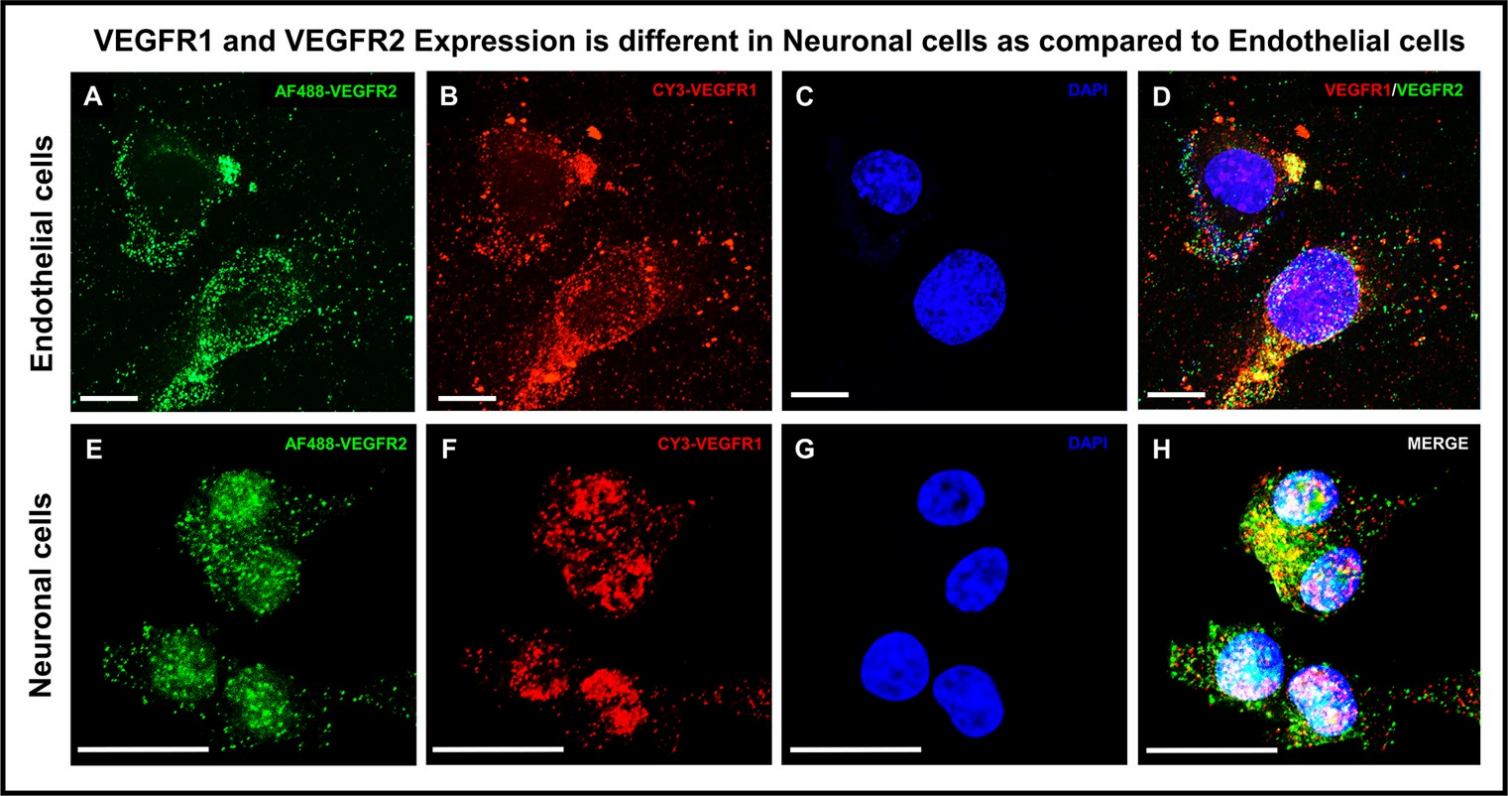
Confocal immunofluorescence microscopy revealed differential VEGFR expression. **(A–H)** A representative image of VEGFR1 co-localized with VEGFR2 in PC12 neuronal and mouse venous endothelial cells. Receptor expression was observed in both, endothelial and neuronal cells with distribution being more nuclear in neuronal cells as compared to endothelial cells. **(A and E)** VEGFR2 immunostaining revealed distinct receptor expression in endothelial and neuronal cells. **(B and F)** VEGFR1 surface immunostaining revealed punctate receptor expression in endothelial and neuronal cells. **(C and G)** DAPI nuclear staining. **(D and H)** Merging of the channels revealed co-expression of both receptors VEGFR1 and VEGFR2 in neuronal and endothelial cells. Scale bar, 10μm.

Fluorescence Immunostaining of VEGFR1 and VEGFR2 was performed in Mouse Venous Endothelial cells (Fig 1A–1D) and PC12 Neuronal cells (Fig 1E–1H). Confocal visualization demonstrated the presence of VEGFR1 and VEGFR2 in both cell types (Fig 1A–1H). The pattern of VEGFR1 and R2 receptor distribution, however, was different in neuronal cells as compared to endothelial cells. VEGFR1 and VEGFR2 distribution in endothelial cells was more peripheral and cytoplasmic in nature (Fig 1 Panel A, B and D) as compared to that seen in neuronal cells (Fig 1 Panel E, F and H). However, both VEGFR1 and VEGFR2 distribution in neuronal cells were more nuclear vis-à-vis endothelial cells. “S1 Fig” clearly shows that after siRNA-mediated VEGFR1 knockdown, VEGFR1 immunostaining revealed little to no expression of VEGFR1 in neuronal and endothelial cells, confirming the specificity of the VEGFR1 antibody used in our studies.

### VEGFR1 and VEGFR2 protein levels in neuronal cells are different from those in endothelial cells

**Fig 2:**
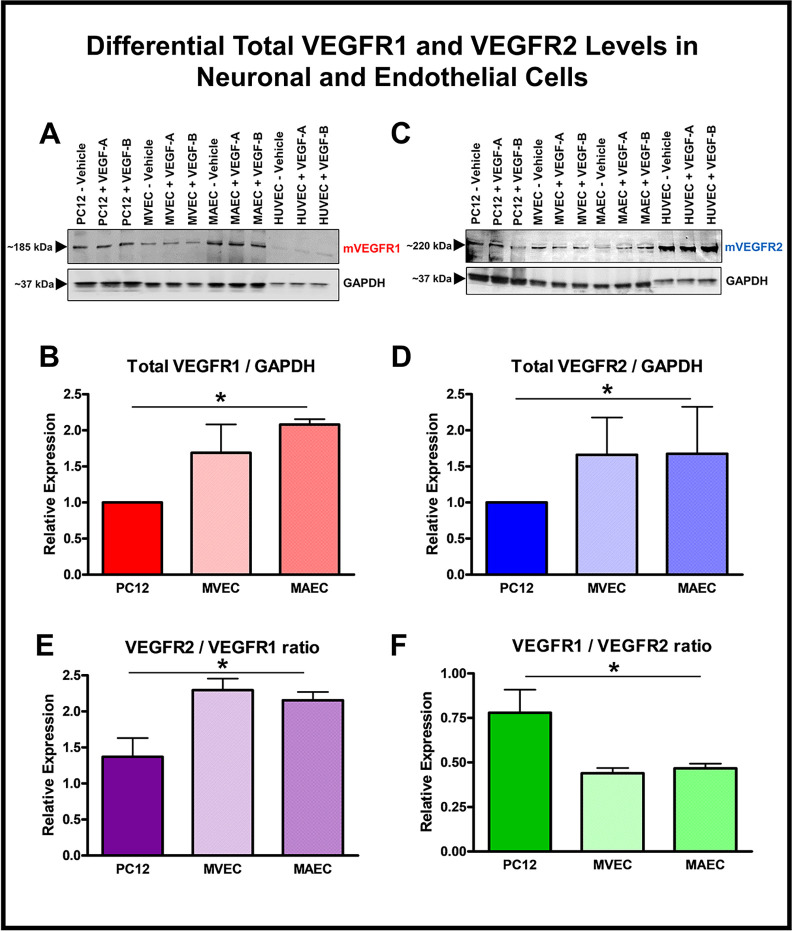
VEGFR1 and VEGFR2 protein expression was different in neuronal versus endothelial cells. **(A and C)** Representative images of immunoblots probed with anti-VEGFR1 (Panel A) or anti-VEGFR2 antibody (Panel C) showed differences in protein levels between endothelial and neuronal cells as determined by band intensity. HUVECs were used as positive controls **(B and D)** Fold increase in VEGFR1 (Panel B) and VEGFR2 (Panel D) expression was observed in both mouse venous (MVEC; VEGFR1-Red Striped Bar; VEGFR2-Blue Striped Bar) and aortic (MAEC; VEGFR1-Red Dotted bar; VEGFR2-Blue Dotted Bar) endothelial cells compared to PC12 neuronal cells (VEGFR1-Red Solid Bar; VEGFR2-Blue Solid Bar). GAPDH was used as a loading control; Fold change values are Mean ± SEM; n = 3; * P < 0.05 for relative VEGFR1 and VEGFR2 levels in endothelial vs neuronal cells **(E)** Relative VEGFR2 / VEGFR1 ratios were higher in both neuronal and endothelial cells (PC12-Violet Solid Bar; MVEC-violet striped bar; MAEC-violet dotted bar). Relative receptor ratios are Mean ± SEM; n = 3; * P < 0.05 for VEGFR2 / VEGFR1 ratio in endothelial cells vs neuronal cells **(F)** Relative VEGFR1 / VEGFR2 ratios were lower in both neuronal and endothelial cells (PC12-Green Solid Bar; MVEC-Green striped bar; MAEC-Green dotted bar). Relative receptor ratios are Mean ± SEM; n = 3; * P < 0.05 for VEGFR1 / VEGFR2 ratio in endothelial vs neuronal cells.

VEGF receptors though structurally similar in different cell populations may have differences in total levels and cellular distribution which may in turn influence homo- and/or heterodimerization and subsequent downstream signaling pathways. Since we already qualitatively demonstrated the existence of VEGFR1 and VEGFR2 by confocal immunofluorescence staining (Fig 1A–1H), we further explored quantitative methodologies to measure receptor expression at the protein level. We evaluated total protein expression of VEGFR1 and VEGFR2 in neuronal cells and compared that to levels in endothelial cells using SDS-PAGE Western blot immunoassays. Total protein extracted from PC12 neuronal, MVEC, MAEC and HUVEC (endothelial cell control) cell lysates when probed with antibodies specific for either VEGFR1 or VEGFR2 demonstrated differential VEGF receptor distribution (Fig 2A–2D). VEGFR expression within a specific-cell population was independent of acute treatment with growth factors, either VEGF-A or VEGF-B (Fig 2A–2D). The ratios of relative levels of VEGFR1 to VEGFR2 (relative band densities to GAPDH) and VEGFR2 to VEGFR1 for neuronal and endothelial cells are also represented (Fig 2E and 2F). A higher ratio of relative levels of VEGFR2 / VEGFR1 in endothelial and neuronal cells indicating higher concentration of total VEGFR2 receptors as compared to total VEGFR1 receptors is in congruence with the published literature. PC12 neuronal cells demonstrated a slightly higher VEGFR1 / VEGFR2 ratio as compared to endothelial cells. However, one should note that all these values are relative and not absolute levels of VEGFR2 and VEGFR1 total protein.

Total VEGFR1 protein levels measured as a ratio of VEGFR1 protein band intensity to that of loading control, GAPDH were found to be 0.8447 ± 0.0257 A.U. (arbitrary units; Mean ± SEM; n = 3) in PC12 neuronal cells as compared to 1.276 ± 0.1982 A.U. (arbitrary units; Mean ± SEM; n = 3) in MVEC and 1.742 ± 0.1817 A.U. in MAEC (arbitrary units; Mean ± SEM; n = 3). Similarly, total VEGFR2 protein expression was found to be lower in PC12 neuronal cells (0.641 ± 0.003 A.U; arbitrary units; Mean ± SEM; n = 3) as compared to levels in MVEC (0.8037 ± 0.06809 A.U; arbitrary units; Mean ± SEM; n = 3) and MAEC (0.6890 ± 0.1180 A.U; arbitrary units; Mean ± SEM; n = 3) respectively. Fig 2B shows a relative VEGFR1 protein expression of 1.690 ± 0.3925 (Mean ± SEM; n = 3) and 2.081 ± 0.0745 (Mean ± SEM; n = 3) in MVEC and MAEC respectively in relation to PC12 neuronal cells and Fig 2C shows a relative VEGFR2 protein expression of 1.661 ± 0.5181 (Mean ± SEM; n = 3) and 1.676 ± 0.6503 (Mean ± SEM; n = 3) in MVEC and MAEC respectively in relation to PC12 neuronal cells. A one-way ANOVA between the 3 groups of cells was found to be significant for total VEGFR1 (P = 0.018) and total VEGFR2 (P = 0.0072) protein expression. Additionally, relative levels or ratios of VEGFR2 to VEGFR1 were found to be lower in PC12 neuronal cells (1.370 ± 0.2614; Mean ± SEM; n = 3) as compared to levels in MVEC (2.295 ± 0.1600; Mean ± SEM; n = 3) and MAEC (2.154 ± 0.1164; Mean ± SEM; n = 3). Relative levels or ratios of VEGFR1 to VEGFR2 were found to be higher in PC12 neuronal cells (0.7793 ± 0.1297; Mean ± SEM; n = 3) as compared to levels in MVEC (0.4398 ± 0.0293; Mean ± SEM; n = 3) and MAEC (0.4671 ± 0.0266; Mean ± SEM; n = 3). A one-way ANOVA between the 3 groups of cells was found to be significant for relative ratios of VEGFR2/VEGFR1 (P = 0.0275) and VEGFR1/VEGFR2 (P = 0.0395).

### Immunoprecipitation studies confirm the presence of VEGFR1-R2 heterodimerization

**Fig 3:**
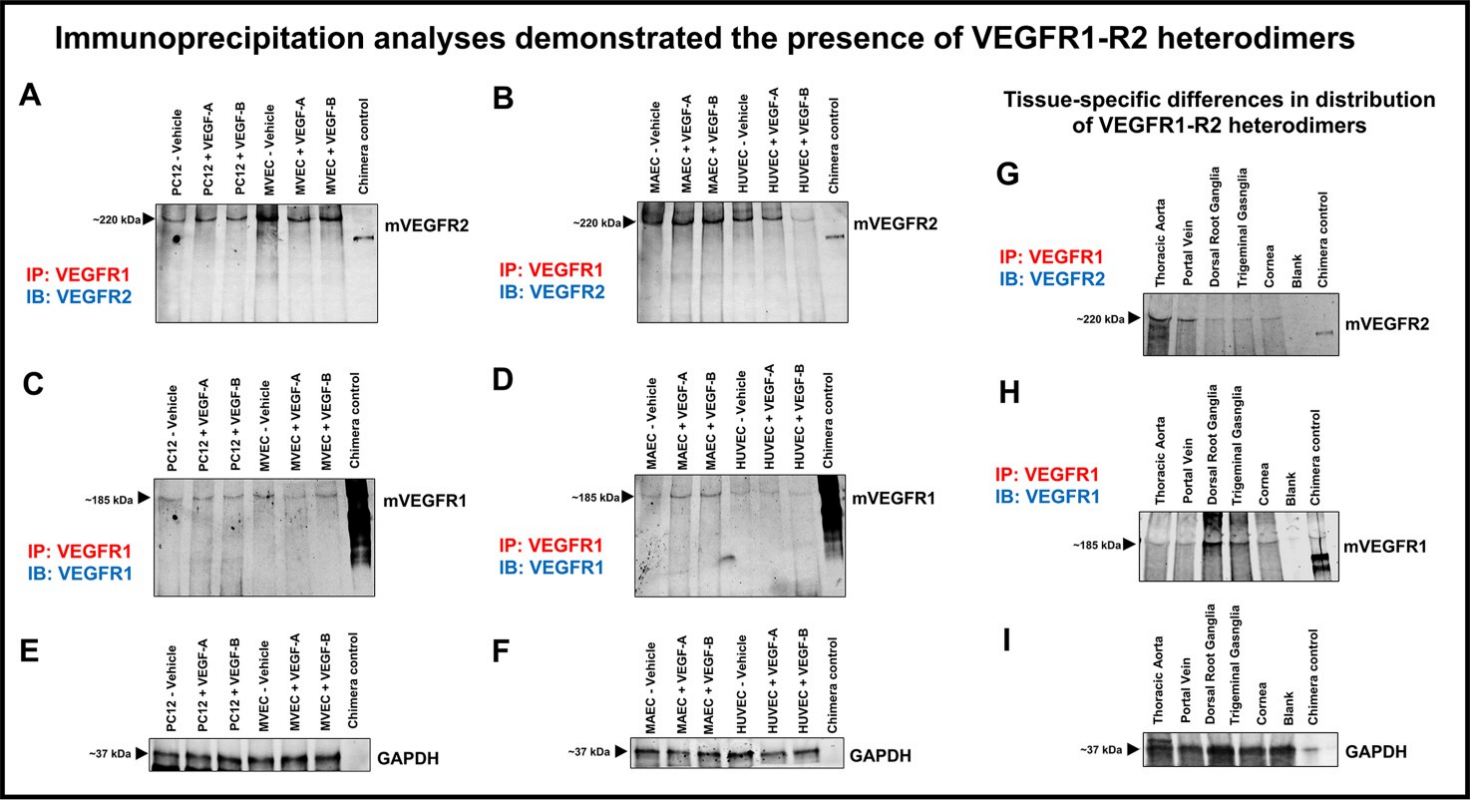
Immunoprecipitation / pulldown assays confirm presence of VEGFR1-R2 heterodimers. **(A)** Immunoblot showing VEGFR2 protein bands in PC12 neuronal and MVEC cell lysates immunoprecipitated with anti-VEGFR1 antibody **(B)** Immunoblot showing VEGFR2 protein bands in MAEC and HUVEC cell lysates immunoprecipitated with VEGFR1 antibody **(C)** Immunoblot from (Panel A), washed and reprobed with anti-VEGFR1 antibody showed VEGFR1 protein bands **(D)** Immunoblot from (Panel B) washed and reprobed with anti-VEGFR1 antibody confirming VEGFR1 pulldown. Cell lysates from PC12 neuronal cells as well as MAEC, MVEC and HUVECs when immunoprecipitated with anti-VEGFR1 antibody demonstrated the existence of VEGFR1-R2 heterodimers when probed with anti-VEGFR2 antibody **(E, F)** Immunoblots showing GAPDH, used as a loading control **(G)** Immunoblot showing VEGFR2 protein bands in tissue lysates immunoprecipitated with anti-VEGFR1 antibody confirm VEGFR1-R2 heterodimers. **(H)** VEGFR1 protein bands in tissue lysates confirmed VEGFR1 pulldown **(I)** GAPDH was used as a loading control.

Cell lysates from PC12 neuronal cells as well as MAEC, MVEC and HUVECs (positive control) were either treated with vehicle or growth factors VEGF-A or VEGF-B. When immunoprecipitated with anti-VEGFR1 antibody, neuronal as well as endothelial cells demonstrated the existence of VEGFR1-R2 heterodimers when probed with anti-VEGFR2 antibody (Figs 3A–3F and “S2”). Pulldowns with anti-VEGFR1 followed by immuno-blotting with anti-VEGFR2 also revealed tissue specific differences in VEGFR1-R2 heterodimers (Fig 3G–3I). Based on relative protein band intensities, VEGFR1-R2 heterodimers were present in higher amounts in thoracic aorta and portal vein as compared to dorsal root ganglia, trigeminal ganglia and cornea. The relative levels of total VEGFR1 were higher in dorsal root ganglia and trigeminal ganglia followed by cornea, portal vein and thoracic aorta. No VEGFR2 protein bands were seen for bead-only control eluates indicating absence of non-specific VEGFR2 binding.

### TIRF microscopy revealed the presence of VEGFR1-R2 heterodimerization in neuronal and endothelial cells

**Fig 4:**
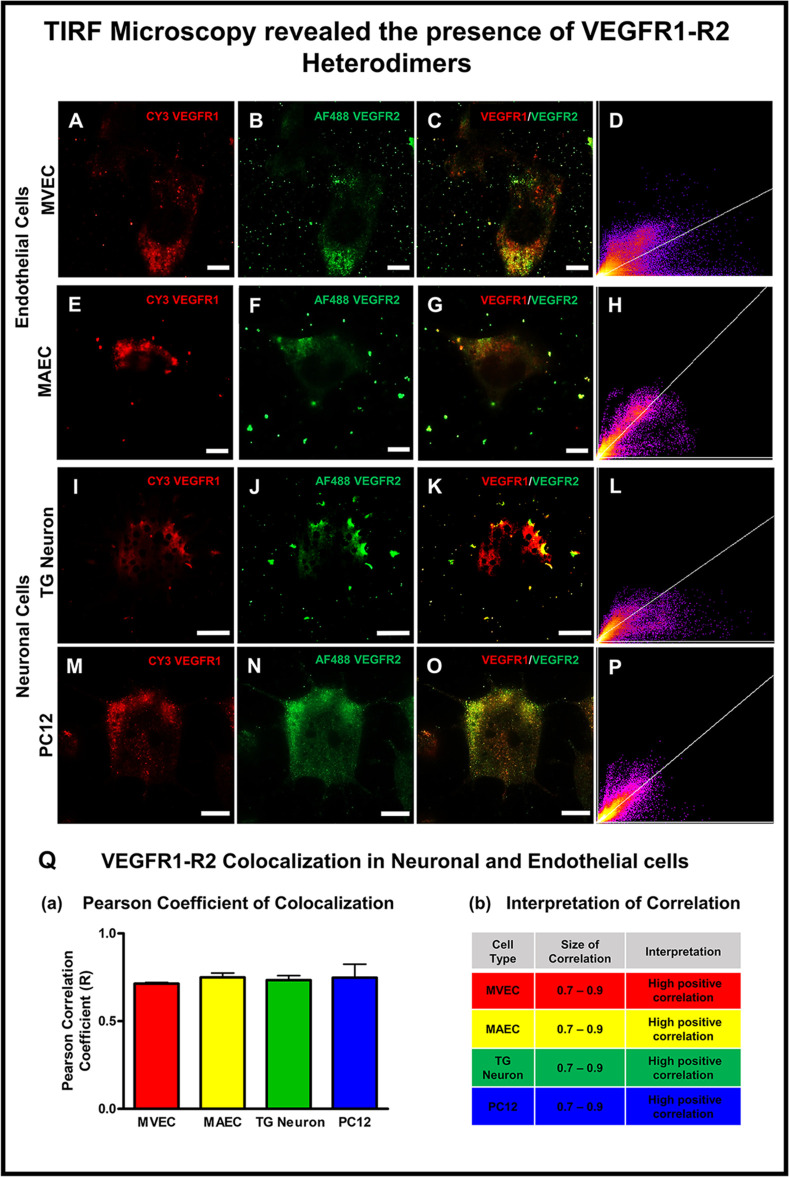
Visualization of VEGFR1-R2 heterodimers by TIRF microscopy. **(A-B)** MVEC showed presence of VEGFR1 and VEGFR2 surface receptors observed as red and green fluorescent signals respectively **(C-D)** Merged image and linear graph showing positive VEGFR1-R2 colocalization **(E-F)** MAEC exhibiting VEGFR1 and VEGFR2 immunostaining **(G-H)** Merged image and receptor colocalization graph confirmed VEGFR1-R2 heterodimer presence **(I-J)** Expression of VEGFR1 and VEGFR2 surface receptors in trigeminal ganglion neuronal cells **(K-L)** Merged image depicting heterodimer presence and colocalization graph confirming the same **(M-N)** PC12 neuronal cells exhibited VEGFR1 (fluorescent red punctate staining) and VEGFR2 (fluorescent green puncta) surface receptors **(O-P)** VEGFR1-R2 heterodimers visualized as yellowish-orange fluorescence and graph showing colocalization between the 2 channels **(Q)** Pearson Correlation Coefficient values were high positive for neuronal and endothelial cells indicating strong colocalization and evidence of receptor heterodimers.

We employed TIRF (Total Internal Reflection Fluorescence) Microscopy to visualize VEGFR1 and VEGFR2 surface receptors within 100 nm of the cell surface layer. This technique helped us locate VEGFR1-R2 heterodimers and obviated the compounding effect of false-positive VEGFR1-R2 colocalization seen in deeper layers of a stacked Z-stack image. Fig 4 depicts TIRF images of endothelial (MVEC; Panels A–D and MAEC; Panels E—H) and neuronal cells (PC12; Panels M—P and Trigeminal ganglion/TG cells; Panels I—L) stained with antibodies against VEGFR1 and VEGFR2. A positive VEGFR1 immunostaining (as evidenced by red fluorescence due to AlexaFluor568-tagged secondary antibody against VEGFR1) was visualized as red punctate dots near the cell surface in neuronal and endothelial cells (Fig 4 Panels A, E, I and M). VEGFR2 surface receptor expression was observed as green fluorescent dots (anti-VEGFR2 antibody followed by AlexaFluor488-tagged secondary antibody) at the cell surface in neuronal and endothelial cells (Fig 4 Panels B, F, J and N). Merged images of VEGFR1 and VEGFR2 immunostaining revealed presence of VEGFR1-R2 heterodimers observed as yellowish-orange fluorescence (Fig 4 Panels C, G, K and O). TIRF Microscopy revealed presence of VEGFR1-R2 heterodimer in trigeminal ganglion and PC12 neuronal cells. NIH Image J Colocalization Threshold and Coloc2 plugins were used to calculate the percent VEGFR1-R2 colocalization in the total receptor population. The percent VEGFR1-R2 colocalization in MVEC and MAEC was found to be 2.68 ± 0.72% and 2.16 ± 0.45% (Mean ± SEM; n = 5) respectively as compared to VEGFR1-R2 colocalization in TG neuronal and PC12 neuronal cells which were found to be 1.27 ± 0.82% and 1.39 ± 0.38% (Mean ± SEM; n = 5) respectively. The Pearson Correlation Coefficient (PCC). The PCC values are +1 for perfect correlation, 0 for no correlation, and -1 for perfect anti-correlation. Both Neuronal and Endothelial cells showed high positive correlation (high extent of VEGFR1-R2 colocalization). MVEC and MAEC showed a high Pearson Correlation Coefficient of 0.7133 ± 0.0067 and 0.7493 ± 0.02549 (Mean ± SEM; n = 5) respectively. Trigeminal Ganglion and PC12 neuronal cells also showed similar Pearson Correlation Coefficient of 0.7333 ± 0.0264 and 0.7480 ± 0.0763 (Mean ± SEM; n = 5) respectively, a strong indicator of VEGFR1-R2 heterodimer presence.

### PLA confirms the presence of receptor homodimers and heterodimers in neuronal cells

**Fig 5:**
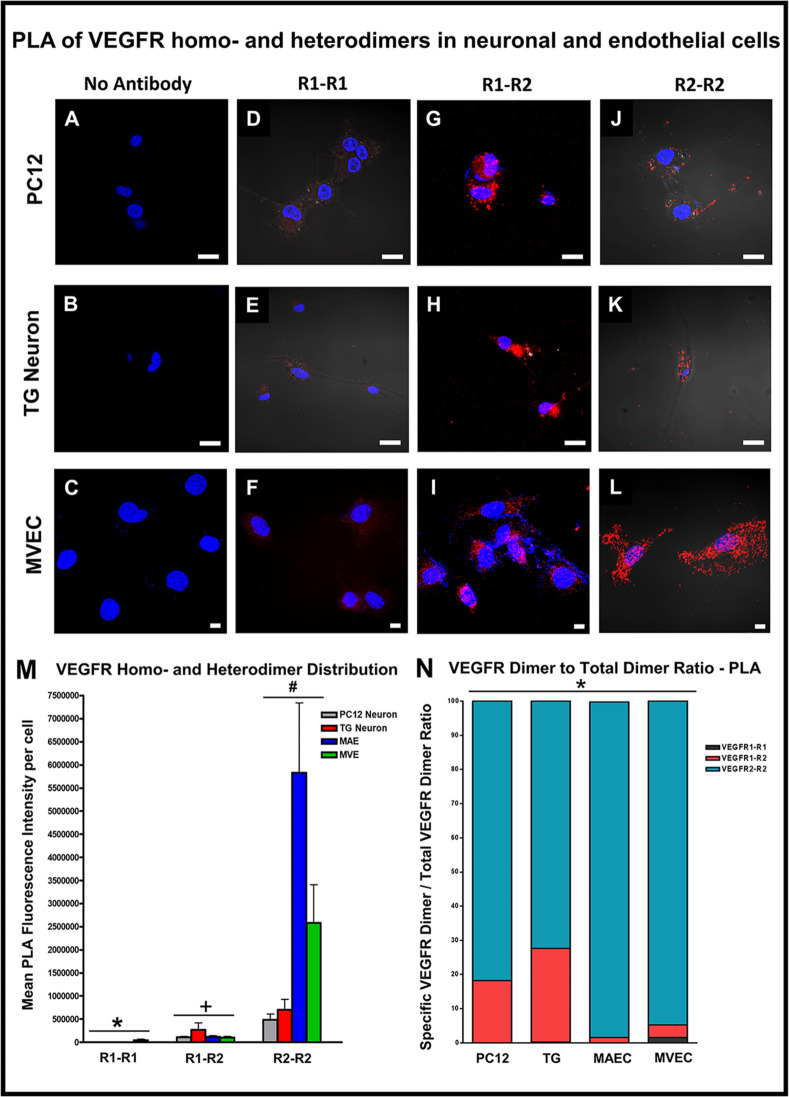
Duolink PLA staining reveals abundant VEGFR2-R2 homodimers and R1-R2 heterodimers. **(A-C)** Negative controls (or no antibody controls) in which neuronal and endothelial cells lacked primary antibody treatment show absence of red PLA staining. **(D-F)** Low levels of VEGFR1-R1 homodimers were found in PC12, TG neuronal and MVECs **(G-I)** R1-R2 heterodimers were visualized as red punctate staining in both types of neuronal cells as well as MVECs **(J-L)** Highest PLA staining corresponding to VEGFR2-R2 homodimers was observed in MVECs as compared to PC12 and TG neuronal cells **(M)** Bar plot based on Mean PLA Fluorescence intensity per cell showed that VEGFR2-R2 homodimers were most abundant in endothelial and neuronal cells *P < 0.05 between dimers in PC12 cells; ^**+**^ P < 0.05 between dimers in MAEC; ^**#**^ P < 0.05 between dimers in MVEC. **(N)** Vertical stacked bar chart based on ratio of VEGFR dimer distribution to total number of dimers showed that neuronal cells had higher expression of VEGFR1-R2 heterodimers as compared to that in endothelial cells. All data are shown as Mean ± SEM (n = 3); * P < 0.05 vs R1-R1 homodimers.

In order to characterize the presence and distribution of VEGFR1 and VEGFR2 homodimers and heterodimers in neuronal cells and endothelial cells we used a novel Duolink® Proximity Ligation Assay. This is based on the proximity ligation assay (PLA) principle, and combines the specificity of secondary antibodies with the sensitivity afforded by rolling circle amplification to detect endogenous proteins in fixed cells and tissues. A pair of oligonucleotide labeled antibodies (PLA probes) generates an amplified signal only when the probes are in close proximity (< 40nm). In order to detect VEGFR1-R2 heterodimers, we used the rat monoclonal anti-mVEGFR1 antibody directly conjugated to DUO92010 Probemaker MINUS probe and the rabbit polyclonal anti-mVEGFR2 antibody with a DUO92002 In Situ PLA anti-rabbit PLUS probe. For detection of VEGFR1-R1 (or R1-R1) homodimers, we used rat monoclonal anti-mVEGFR1 antibody conjugated to a DUO92010 Probemaker MINUS probe and another rat monoclonal anti-mVEGFR1 antibody conjugated to a DUO92009 Probemaker PLUS probe and for VEGFR2-R2 (or R2-R2) homodimer detection we used the rabbit polyclonal anti-mVEGFR2 antibody conjugated to a DUO92010 Probemaker MINUS probe and another rabbit polyclonal anti-mVEGFR2 antibody conjugated to a DUO92009 Probemaker PLUS probe. Duolink PLA staining for VEGFR1-R1 homodimers showed faint punctate staining indicating very low levels of VEGFR1-R1 homodimers in both neuronal (TG neuronal: 2.196 x 10^3^ ± 1.891 x 10^3^; Mean ± SEM; n = 3) as well as endothelial cells (MVEC: 4.332 x 10^4^ ± 2.29 x 10^3^; Mean ± SEM; n = 3; Fig 5D–5F) as compared to VEGFR2-R2 homodimers (PC12: 4.885 x 10^5^ ± 1.21 x 10^5^; TG neuronal: 7.062 x 10^5^ ± 2.264 x 10^5^; MAEC: 5.833 x 10^6^ ± 1.511 x 10^6^; MVEC: 2.578 x 10^6^ ± 8.325 x 10^5^; Mean ± SEM; n = 3; Fig 5J–5L) and VEGFR1-R2 (or R1-R2) heterodimers (PC12: 1.087 x 10^5^ ± 1.75 x 10^4^; TG neuronal: 2.68 x 10^5^ ± 1.54 x 10^5^; MAEC: 1.204 x 10^5^ ± 2.053 x 10^4^; MVEC: 9.9613 x 10^4^ ± 2.4761 x 10^4^; Mean ± SEM; n = 3; Fig 5G–5I). Duolink PLA staining for VEGFR1-R2 heterodimers showed distinct red fluorescent punctate staining indicating much higher levels of VEGFR1-R2 heterodimers as compared to R1-R1 homodimers in both neuronal (Fig 5G) and TG Neuronal (Fig 5H) as well as endothelial cells (Fig 5I). Duolink PLA staining for VEGFR2-R2 homodimers showed very bright discrete red fluorescent punctate spots of VEGFR2-R2 homodimers. Endothelial cells (Fig 5L) showed higher levels of VEGFR2-R2 homodimers as compared to PC12 neuronal cells (Fig 5J) and TG Neuronal (Fig 5K) cells. Both Neuronal as well as Endothelial cells showed higher expression of VEGFR2-R2 homodimers as compared to VEGFR1-R2 heterodimers and VEGFR1-R1 homodimers. A vertical interleaved bar plot (Fig 5M) shows that R2-R2 homodimer expression levels were the highest, followed by R1-R2 heterodimer and then R1-R1 homodimer expression in both neuronal and endothelial cells. A one-way ANOVA for Mean PLA Fluorescence Intensity per cell between the 3 groups of receptor dimers was found to be significant in PC12 cells (P = 0.0063), MAEC (P = 0.0050) and MVEC (P = 0.0154). A vertical stacked bar plot (Fig 5N) shows that neuronal cells showed higher levels of VEGFR1-R2 heterodimers as compared to endothelial cells. Endothelial cells showed higher VEGFR2-R2 homodimers as compared to neuronal cells. VEGFR1-R1 homodimer levels were extremely low in both neuronal and endothelial cells. A one-way ANOVA for % VEGFR Dimer / total VEGFR Dimer ratio between neuronal and endothelial cells was found to be significant (P = 0.0001). A paired t-test of % VEGFR Dimer / total VEGFR Dimer ratio was significant between VEGFR1-R1 and VEGFR2-R2 homodimers (P = 0.0136). Negative controls without primary antibodies (Fig 5A–5C) as well as “S3 Fig” in which neuronal and endothelial cells were treated without primary antibodies showed absence of red PLA staining.

## Discussion

Our study yielded the following important findings: (1) We confirmed the existence of VEGFR1-R2 heterodimers in endothelial cells and for the first time demonstrated their presence in neuronal cells using confocal fluorescence microscopy and TIRF microscopy (2) Tissue-wide expression profiling of VEGFR1-R2 heterodimers by immunoprecipitation showed differential tissue distribution. (3) Immunoprecipitation with specific VEGF receptors revealed complexes of VEGFR1-R2 heterodimers for the first time in neuronal cells (4) VEGFR2-R2 homodimers were expressed in higher levels in endothelial cells as well as neuronal cells as compared to VEGFR1-R1 homodimer expression which was low in both cell types (5) VEGFR1-R2 heterodimer presence in relation to total VEGFR dimers was higher in neuronal cells versus endothelial cells

Peripheral nerve injury (PNI) causes loss of sensory and/or motor function and nerve repair and regeneration requires a complex interplay of cellular and molecular processes [22]. There is increasing evidence of similarities in the molecules and signaling pathways responsible for neurovascular development and repair. Nerves and arteries share ligand and receptor pairs, and in many systems there is a congruence of nerve and vessel patterning. In some models, nerves appear to pattern vessels, in others the vessels pattern the nerves [23]. The cornea is a highly metabolically active tissue but is devoid of blood vessels despite having the highest nerve density known. This disconnect in the absence of vessels and presence of nerves in the cornea is noteworthy and makes the cornea a unique model for the study of both vessels and nerves.

Several studies have implicated the VEGF family of growth factors in developmental neurogenesis and pathological nerve regeneration [6–8]. VEGF (Vascular Endothelial Growth Factor) are a family of growth factors which signal through receptor tyrosine kinases. At least 5 members of the VEGF gene family (VEGF-A to–E) have been described of which VEGF-A is the best studied member and is responsible for most of the angiogenic and neurogenic actions of VEGF. VEGF-B has a role in neuroprotection, VEGF-C in lymphangiogenesis, VEGF-D in wound healing, and VEGF-E in viral signaling [20, 24].

Elevated expression of VEGF-A and VEGF-B during peripheral nerve injury have been demonstrated in several studies [6, 25, 26]. Previous studies from our lab have shown that VEGF-A, a pro-angiogenic factor, stimulates nerve regeneration and is involved in functional recovery of injured peripheral nerves [12]. Recently we have also demonstrated VEGF-B to be a potent player in the neuroregenerative process. Our studies showed that VEGF-B treatment restored anatomic and functional re-innervation in injured corneal peripheral nerves via increased nerve regeneration, enhanced trophic functions and recovery of sensation independent of any vascular involvement [5].

VEGF types and their isoforms have variable affinities for VEGF receptors. VEGF receptor types include VEGFR-1, -2, and -3 which are tyrosine kinase type receptors able to transduce signals upon VEGF binding. Neuropilins-1 and -2 act as co-receptors for VEGFRs (as well as for semaphorins) to modulate the cellular response to VEGF [20]. The angiogenic effects of VEGF seem to rest primarily in activation of VEGFR-2 (KDR, Flk-1), but VEGF-A binds VEGFR-1 (Flt-1) with high affinity as well. Upon VEGF homodimer binding, VEGFRs dimerize, allowing cross-phosphorylation and activation of multiple cell signaling cascades [19, 21]. Presence of receptor homodimers and heterodimers between VEGFR1 and VEGFR2 has been demonstrated in endothelial cells as well as in cell-free systems [27–31] but to date, not much is known about VEGF receptor expression in neurons.

Prior studies from our group demonstrated that the responses of TG neurons to VEGF-A were eliminated by neutralizing antibodies to VEGFR1, VEGFR2, or NP1 [12]. The absolute requirement for all three receptor types was surprising given that VEGF-A should have been able to signal through VEGFR2 even in the presence of VEGFR1 blocking. It is unclear whether concomitant signaling through VEGFR1 and VEGFR2 homodimers complexed with NP1 is necessary, or whether VEGF-A signals through VEGFR1:VEGFR2 heterodimers which were blocked by either VEGR1 or VEGFR2 antibodies. VEGF-B is known to bind exclusively to VEGFR1 and NP1 complexes, and our blocking antibody data was consistent with these findings [5]. This stimulation of nerve growth appears to be different compared to stimulation of angiogenesis by these same ligands. There are a number of competing hypotheses which might explain these observed differences in VEGF ligand activity on nerves as compared to vessels and one of our leading hypotheses was that nerves and vessels have a different complement of receptor homo- and heterodimers, or a different complement of co-receptors which leads to altered signaling pathway activation.

These differences could be related to receptors types, or could be due to tissue specific differences in receptor number, internalization, or up/downregulation upon exposure to ligands, differences in downstream signaling between ligands and tissues that could be mediated by the above mechanisms or by actual differences in downstream signaling component availability/quantity.

Our current investigations into the subtle differences / similarities between angiogenesis and neurogenesis compared dimer presence and distribution in the endothelial cell group with mouse trigeminal ganglia neurons as well as a PC12 rat neuronal cell line which possesses VEGF responses similar to that seen for primary TG neurons. For our studies on the presence and/or distribution of VEGFR1-R2 homo and heterodimers in endothelial cells, we used MAEC (mouse aortic endothelial cell line) and MVEC (mouse venous endothelial cell line) for our study group and HUVEC (human umbilical venous endothelial cell line) as our control group. Our confocal and TIRF immunofluorescence data strongly confirmed the existence of VEGFR1-R2 heterodimers in endothelial cells and for the first time in neuronal cells. Furthermore, we found that cell lysates from PC12 neuronal cells as well as MAE and MVE cells when immunoprecipitated with anti-VEGFR1 antibody, demonstrated the existence of VEGFR1-R2 heterodimers when probed with anti-VEGFR2 antibody thus validating the existence of VEGFR1-R2 heterodimers. Our findings corroborate with earlier work demonstrating heterodimerization of receptor subunits in G-protein-coupled receptors and Receptor-Tyrosine Kinase systems involving epidermal growth factor (EGF) and platelet-derived growth factor (PDGF) [32–36].

In the current study, tissue-wide expression profiling of VEGFR1-R2 heterodimers by immunoprecipitation showed differences in tissue-specific expression suggesting variations in receptor homodimer and heterodimer availability as well as in existence of preassembled VEGFR1 − 2 heterodimer receptors in neuronal and endothelial cells. This phenomenon has also been observed by other groups in endothelial cells [37, 38]. Computer modeling studies have demonstrated the predominance of heterodimers when cells express both receptors VEGFR1 and R2 [31, 39]. There is further credence to this possibility since VEGFR1 is approximately 10-fold lesser in abundance compared to VEGFR2 thus suggesting that majority of the endothelial cells exist as heterodimers [40]. Our TIRF microscopy data showed that neuronal and endothelial cells demonstrated a high presence of VEGFR2-R2 homodimerization, followed by VEGFR1-R2 heterodimerization and low levels of VEGFR1-R1 homodimerization.

Using a novel dimeric ligand comprising of one VEGFR-2-specific monomer (VEGF-E) and a VEGFR-1-specific monomer (PlGF-1), Cudmore et al. have shown that in endothelial cells, VEGFR1-R2 activation mediates VEGFR phosphorylation, endothelial cell migration, sustained in vitro tube formation and vasorelaxation via the nitric oxide pathway and that VEGFR1-R2 activation does not mediate proliferation or elicit endothelial tissue factor production, confirming that these functions are probably controlled by VEGFR2-R2 homodimers [31, 41]. They further concluded that VEGFR1-R2 heterodimers negatively regulate pro-angiogenic and proliferative function mediated by VEGFR2-R2 homodimers, in turn regulating angiogenesis and maintaining endothelial cell homeostasis.

In our current study, the reasons for the differences in VEGFR1-R1 and VEGFR2-R2 homodimers and VEGFR1-R2 heterodimers in neuronal cells are not very clear and the possible involvement of multiple factors and signaling pathways affecting nerve regeneration needs to be evaluated further. Nevertheless, our findings of higher expression of VEGFR1-R2 heterodimers as compared to that in endothelial cells suggests the importance of VEGFR1-R2 heterodimers in neuronal cell regulation and homeostasis. Our data also emphasizes the possibility that previously unreported VEGFR combinations may be important in regulation of nerve regeneration in which ligands may function predominantly though VEGFR1-R2 heterodimers. Further investigation into the mechanisms of specific VEGF signaling during nerve regeneration will allow for the development of therapeutic agents specifically tailored to treat the myriad corneal diseases related to neural injury.

In conclusion, to our knowledge this is the first report to characterize VEGFR1-R2 heterodimers as well as VEGFR1-R1 and VEGFR2-R2 homodimers in neuronal cells with abundance levels being different from those demonstrated in endothelial cells. Further studies need to be carried out in order to evaluate and characterize the functional significance of VEGFR1-R2 heterodimers in regenerating corneal nerves. Our findings may set the groundwork for the development of VEGF related therapies for peripheral nerve injury.

## Supporting information

S1 Fig siVEGFR1-treated neuronal and endothelial cells do not show VEGFR1 immunofluorescence as visualized by confocal microscopy. PC12, MVEC, HUVEC and TG neuronal cells were treated with either siRNA against VEGFR1 (VEGFR1 siRNA; 200 nM; sc-35395; Santa Cruz Biotech) or Control siRNA (sc-36869; 200 nM: Santa Cruz Biotech) as per manufacturer’s instructions. Immunostaining and immunofluorescence microscopy is as mentioned in “Materials and Methods” section. (A, E, I, M) VEGFR1 surface immunostaining revealed punctate receptor expression in control siRNA treated endothelial (HUVEC, MVEC) and neuronal cells (PC12 neuronal, TG neuronal). (B, F, J, N) DAPI nuclear staining. (C, G, K, O) siVEGFR1 treatment caused loss of VEGFR1 staining in endothelial (HUVEC, MVEC) and neuronal cells (PC12 neuronal, TG neuronal) due to VEGFR1 knockdown proving that the VEGFR1 antibody is specific for all the cell lines used in the study. (D, H, L, P) DAPI nuclear staining. Scale bar, 10μm.
(PDF)

S2 Fig Immunoblots containing VEGFR1-Immunoprecipitated Eluates showing presence of VEGFR1-R2 heterodimers and absence of non-specific VEGFR2 binding in the bead-only control eluates (A) Immunoblot probed with VEGFR2 antibody after IP with VEGFR1. Lanes 1–4 depict Bead Control IP Eluates from PC12, MAEC, MVEC and HUVEC. No VEGFR2 protein band was seen in these lanes indicating absence of non-specific binding whereas Lanes 6–10 show distinct VEGFR2 protein bands indicating the presence of VEGFR1-R2 heterodimers (B) Immunoblot probed with VEGFR1 antibody after IP with VEGFR1. No VEGFR1 protein band was seen in these lanes indicating absence of non-specific binding whereas Lanes 6–10 show distinct VEGFR1 protein bands confirming IP with VEGFR1.
(PDF)

S3 Fig Duolink PLA staining is negative in the absence of primary antibodies (A-C) PC12 Negative controls or (D-F) MAEC negative controls or (G-I) MVEC negative controls without primary antibodies showed absence of red PLA staining indicating the specificity of Duolink PLA staining for the detection of homodimers and heterodimers.
(PDF)

S1 Raw images Original, uncropped and minimally adjusted images supporting all blot and gel results. (PDF)
