# Supplementary figures and images for: VEGF receptor heterodimers and homodimers are differentially expressed in neuronal and endothelial cell types
Source: PLoS One. 2022 Jul 21;17(7):e0269818. doi: 10.1371/journal.pone.0269818 (PMC9302817; doi:10.1371/journal.pone.0269818)

S1 Fig

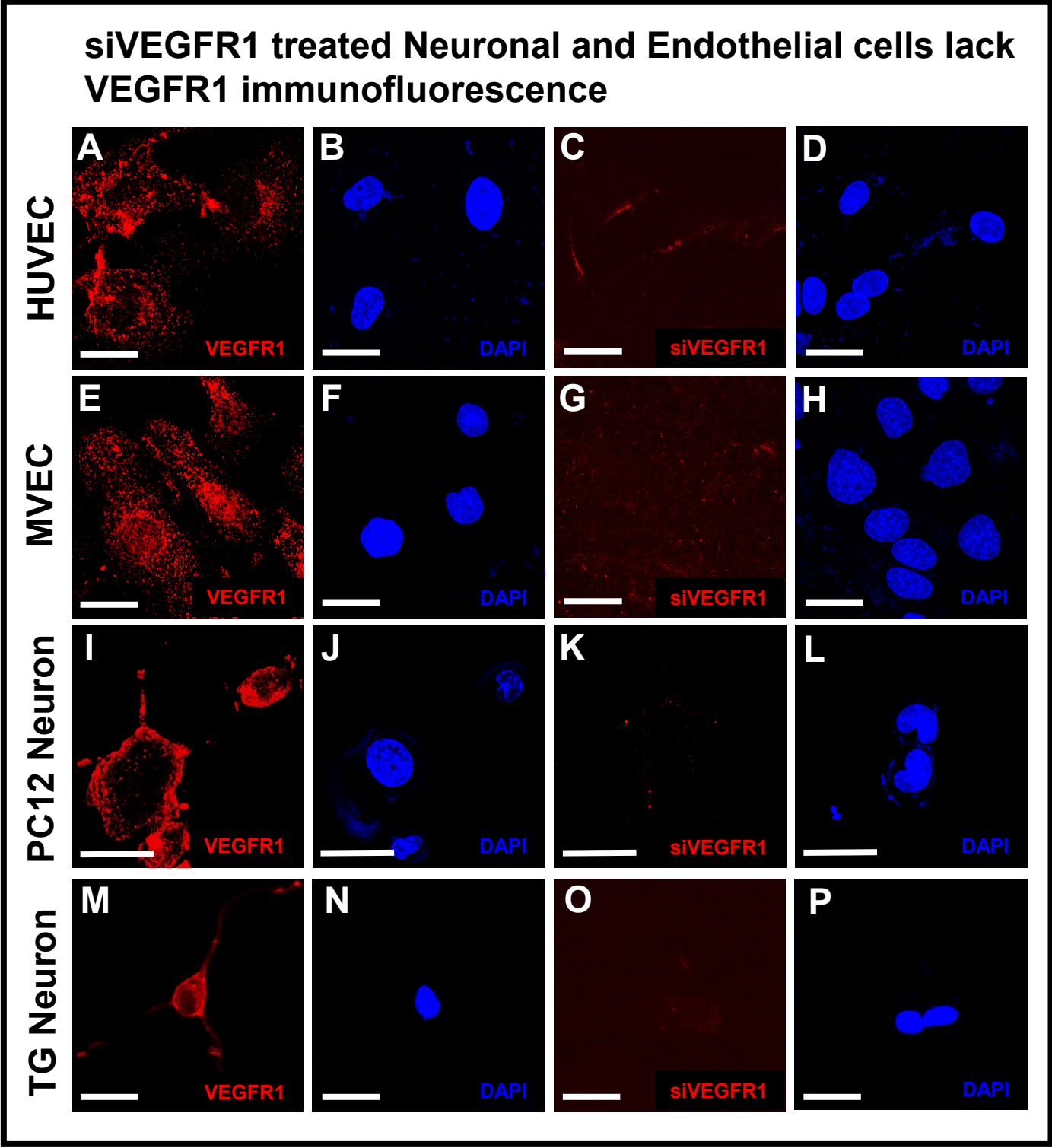

Supplement: S1 Fig — PC12, MVEC, HUVEC and TG neuronal cells were treated with either siRNA against VEGFR1 (VEGFR1 siRNA; 200 nM; sc-35395; Santa Cruz Biotech) or Control siRNA (sc-36869; 200 nM: Santa Cruz Biotech) as per manufacturer’s instructions. Immunostaining and immunofluorescence microscopy is as mentioned in “Materials and Methods” section. (A, E, I, M) VEGFR1 surface immunostaining revealed punctate receptor expression in control siRNA treated endothelial (HUVEC, MVEC) and neuronal cells (PC12 neuronal, TG neuronal). (B, F, J, N) DAPI nuclear staining. (C, G, K, O) siVEGFR1 treatment caused loss of VEGFR1 staining in endothelial (HUVEC, MVEC) and neuronal cells (PC12 neuronal, TG neuronal) due to VEGFR1 knockdown proving that the VEGFR1 antibody is specific for all the cell lines used in the study. (D, H, L, P) DAPI nuclear staining. Scale bar, 10μm. (PDF) [file pone.0269818.s001.pdf]

S2 Fig

**A**

**IP: VEGFR1**  
**IB: VEGFR2**

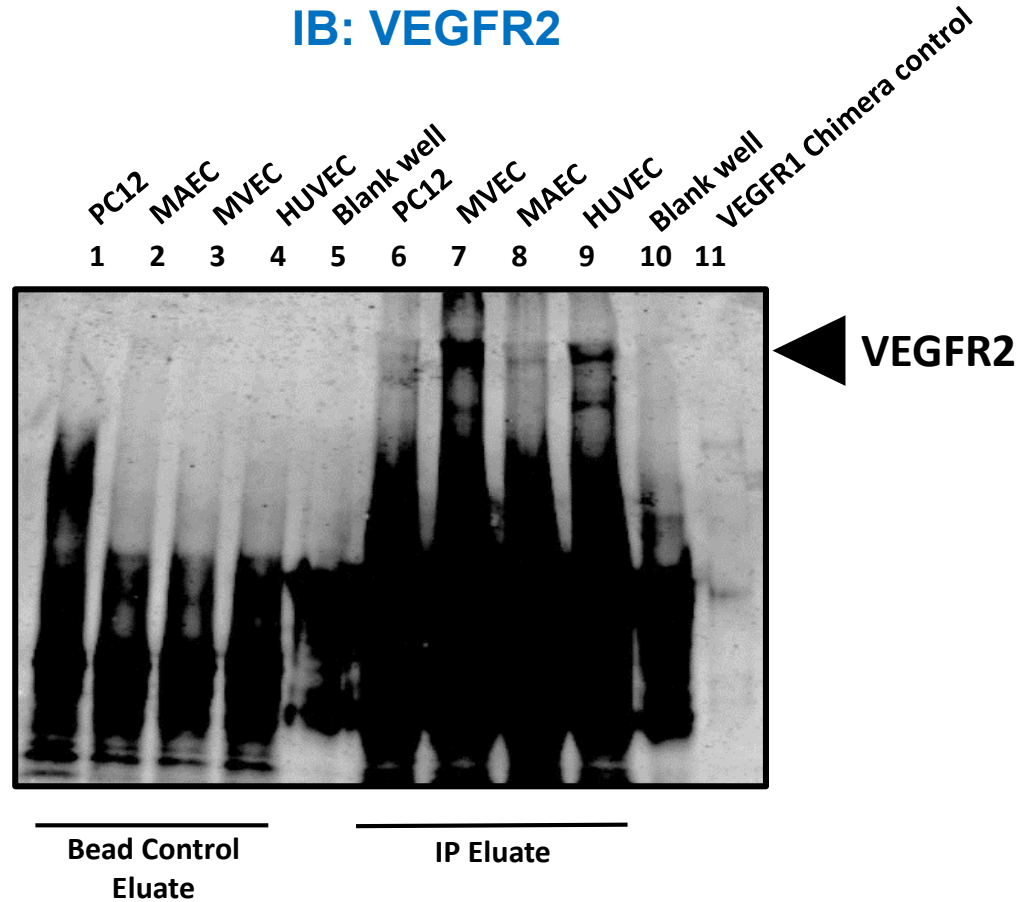

**B**

**IP: VEGFR1**  
**IB: VEGFR1**

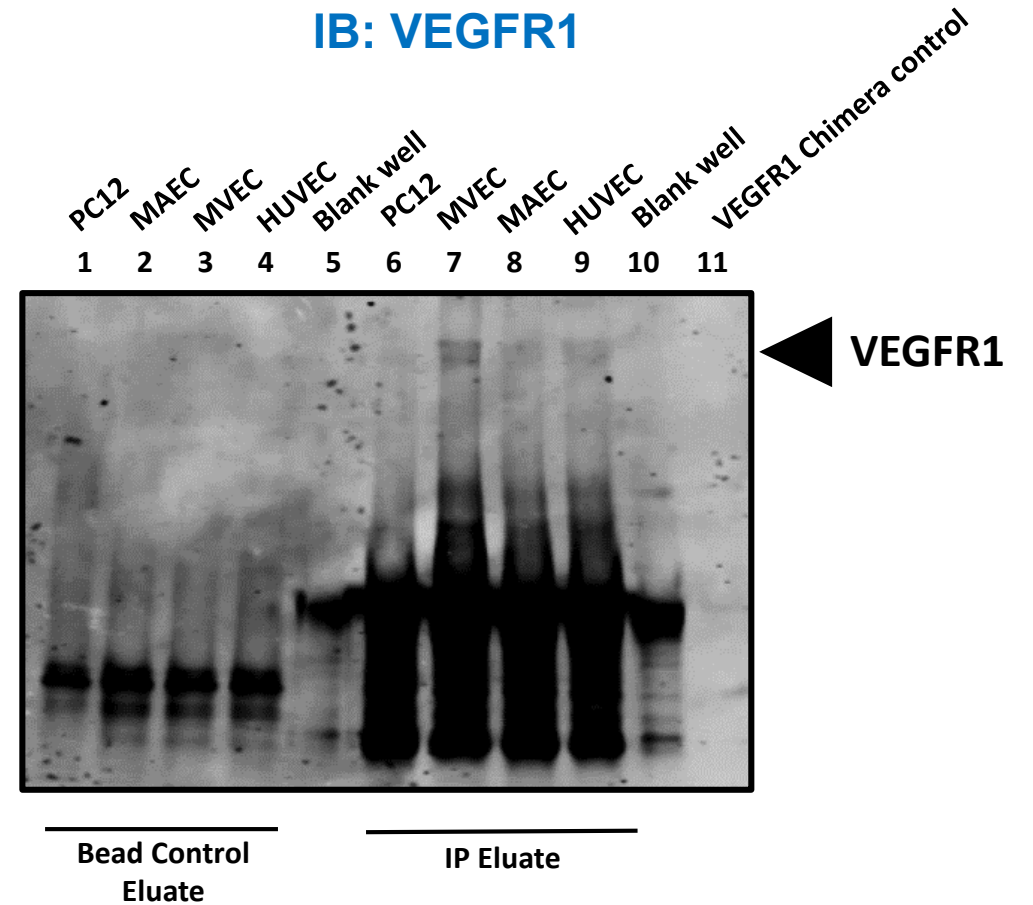

Supplement: S2 Fig — Immunoblots containing VEGFR1-Immunoprecipitated Eluates showing presence of VEGFR1-R2 heterodimers and absence of non-specific VEGFR2 binding in the bead-only control eluates (A) Immunoblot probed with VEGFR2 antibody after IP with VEGFR1. Lanes 1–4 depict Bead Control IP Eluates from PC12, MAEC, MVEC and HUVEC. No VEGFR2 protein band was seen in these lanes indicating absence of non-specific binding whereas Lanes 6–10 show distinct VEGFR2 protein bands indicating the presence of VEGFR1-R2 heterodimers (B) Immunoblot probed with VEGFR1 antibody after IP with VEGFR1. No VEGFR1 protein band was seen in these lanes indicating absence of non-specific binding whereas Lanes 6–10 show distinct VEGFR1 protein bands confirming IP with VEGFR1. (PDF) [file pone.0269818.s002.pdf]
