# Supplementary material for: VEGF receptor heterodimers and homodimers are differentially expressed in neuronal and endothelial cell types
Source: PLoS One. 2022 Jul 21;17(7):e0269818. doi: 10.1371/journal.pone.0269818 (PMC9302817; doi:10.1371/journal.pone.0269818)
Supplement: S3 Fig — Duolink PLA staining is negative in the absence of primary antibodies (A-C) PC12 Negative controls or (D-F) MAEC negative controls or (G-I) MVEC negative controls without primary antibodies showed absence of red PLA staining indicating the specificity of Duolink PLA staining for the detection of homodimers and heterodimers. (PDF) [file pone.0269818.s003.pdf]

S3 Fig

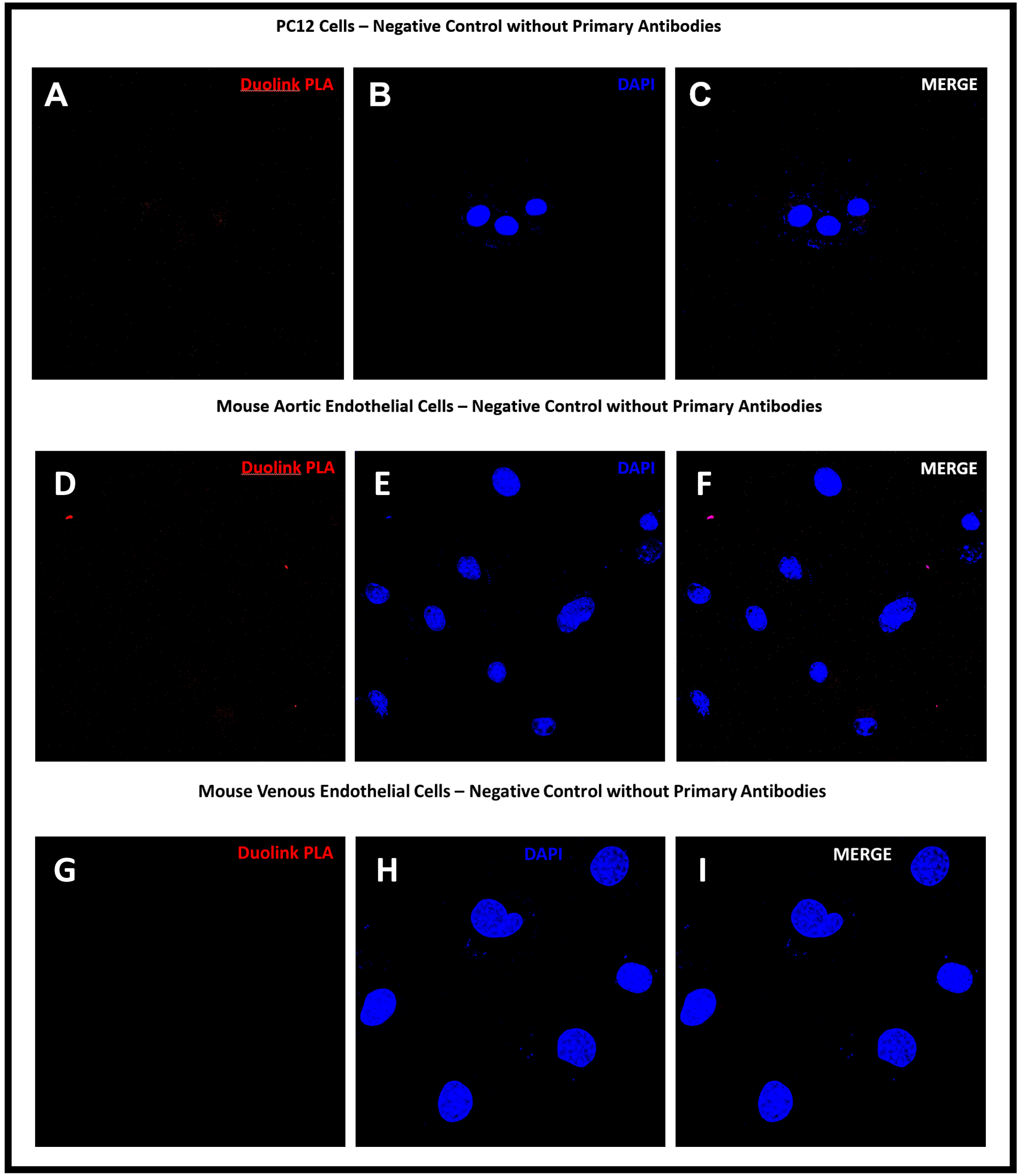
PC12 Cells – Negative Control without Primary Antibodies
A
Duolink PLA
B
DAPI
C
MERGE
Mouse Aortic Endothelial Cells – Negative Control without Primary Antibodies
D
Duolink PLA
E
DAPI
F
MERGE
Mouse Venous Endothelial Cells – Negative Control without Primary Antibodies
G
Duolink PLA
H
DAPI
I
MERGE
