# Supplementary material for: VEGF receptor heterodimers and homodimers are differentially expressed in neuronal and endothelial cell types
Source: PLoS One. 2022 Jul 21;17(7):e0269818. doi: 10.1371/journal.pone.0269818 (PMC9302817; doi:10.1371/journal.pone.0269818)
Supplement: S1 Raw images — (PDF) [file pone.0269818.s004.pdf]

Figure 2 A

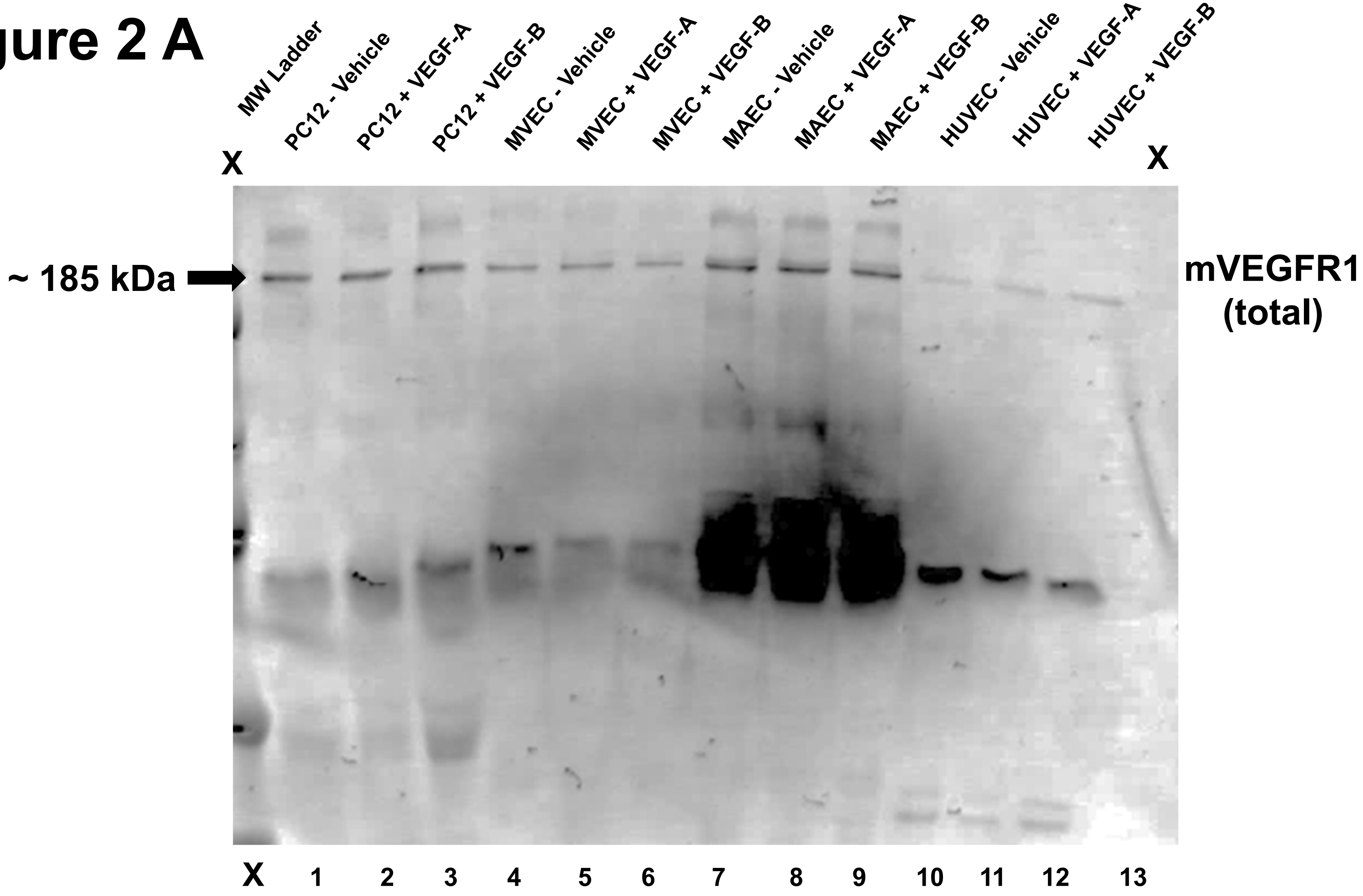
MW Ladder
PC12 - Vehicle
PC12 + VEGF-A
PC12 + VEGF-B
MVEC - Vehicle
MVEC + VEGF-A
MVEC + VEGF-B
MAEC - Vehicle
MAEC + VEGF-A
MAEC + VEGF-B
HUVEC - Vehicle
HUVEC + VEGF-A
HUVEC + VEGF-B
X
X
~ 185 kDa
mVEGFR1
(total)
X
1
2
3
4
5
6
7
8
9
10
11
12
13

Figure 2 A

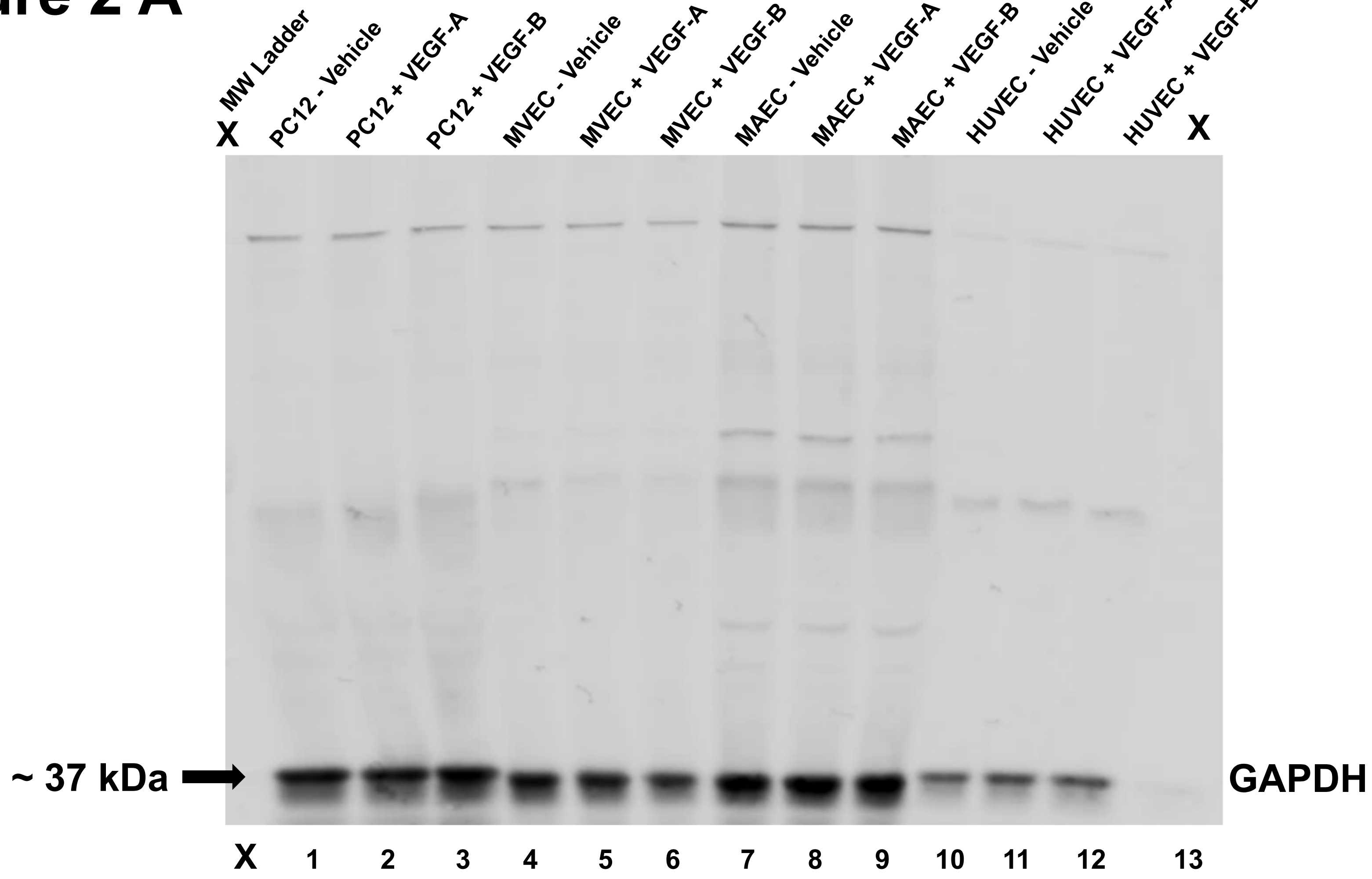
MW Ladder
PC12 - Vehicle
PC12 + VEGF-A
PC12 + VEGF-B
MVEC - Vehicle
MVEC + VEGF-A
MVEC + VEGF-B
MAEC - Vehicle
MAEC + VEGF-A
MAEC + VEGF-B
HUVEC - Vehicle
HUVEC + VEGF-A
HUVEC + VEGF-B
X
X
~ 37 kDa
GAPDH
X
1
2
3
4
5
6
7
8
9
10
11
12
13

Figure 2 C

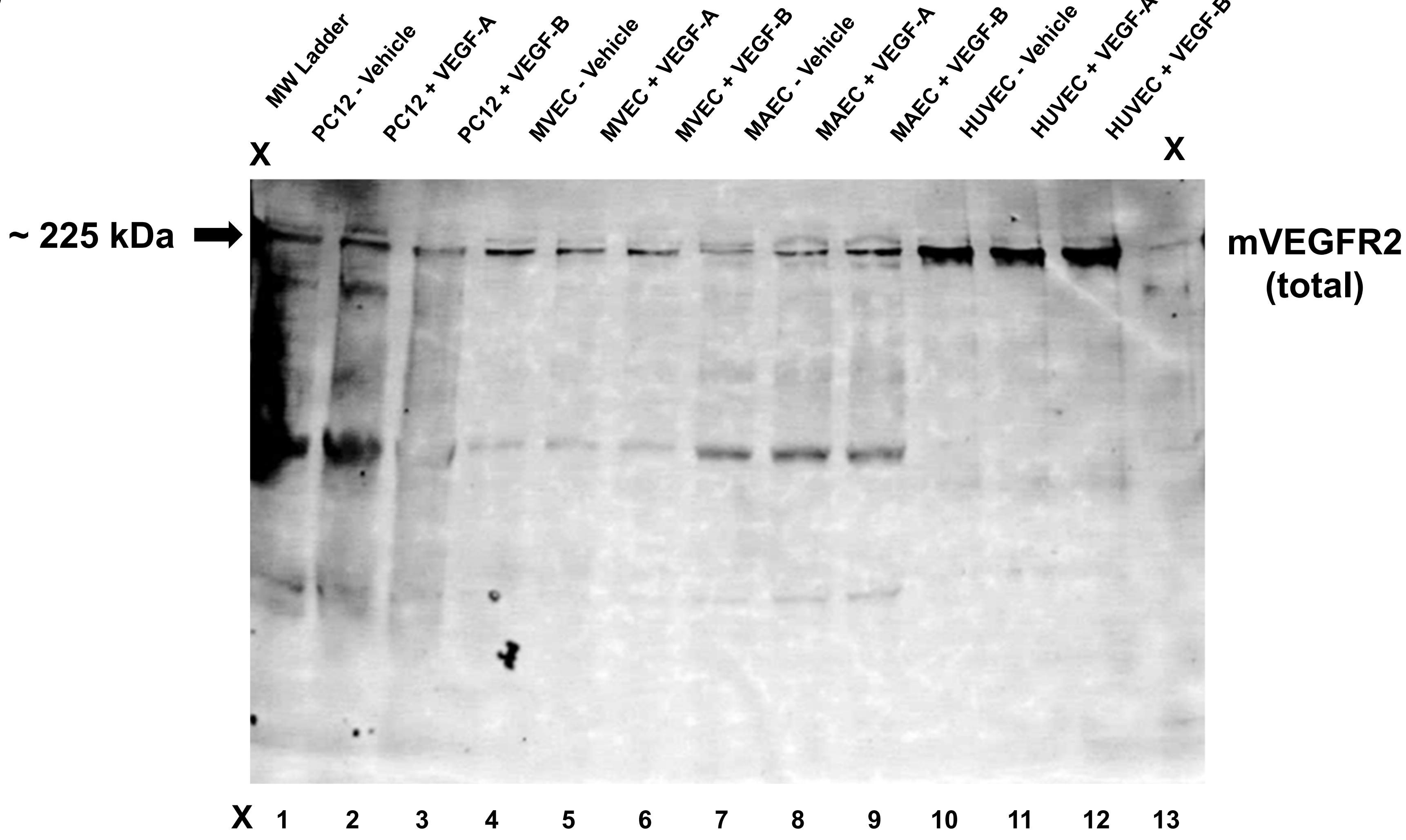
MW Ladder
PC12 - Vehicle
PC12 + VEGF-A
PC12 + VEGF-B
MVEC - Vehicle
MVEC + VEGF-A
MVEC + VEGF-B
MAEC - Vehicle
MAEC + VEGF-A
MAEC + VEGF-B
HUVEC - Vehicle
HUVEC + VEGF-A
HUVEC + VEGF-B
X
X
~ 225 kDa
mVEGFR2
(total)
X 1 2 3 4 5 6 7 8 9 10 11 12 13

Figure 2 C

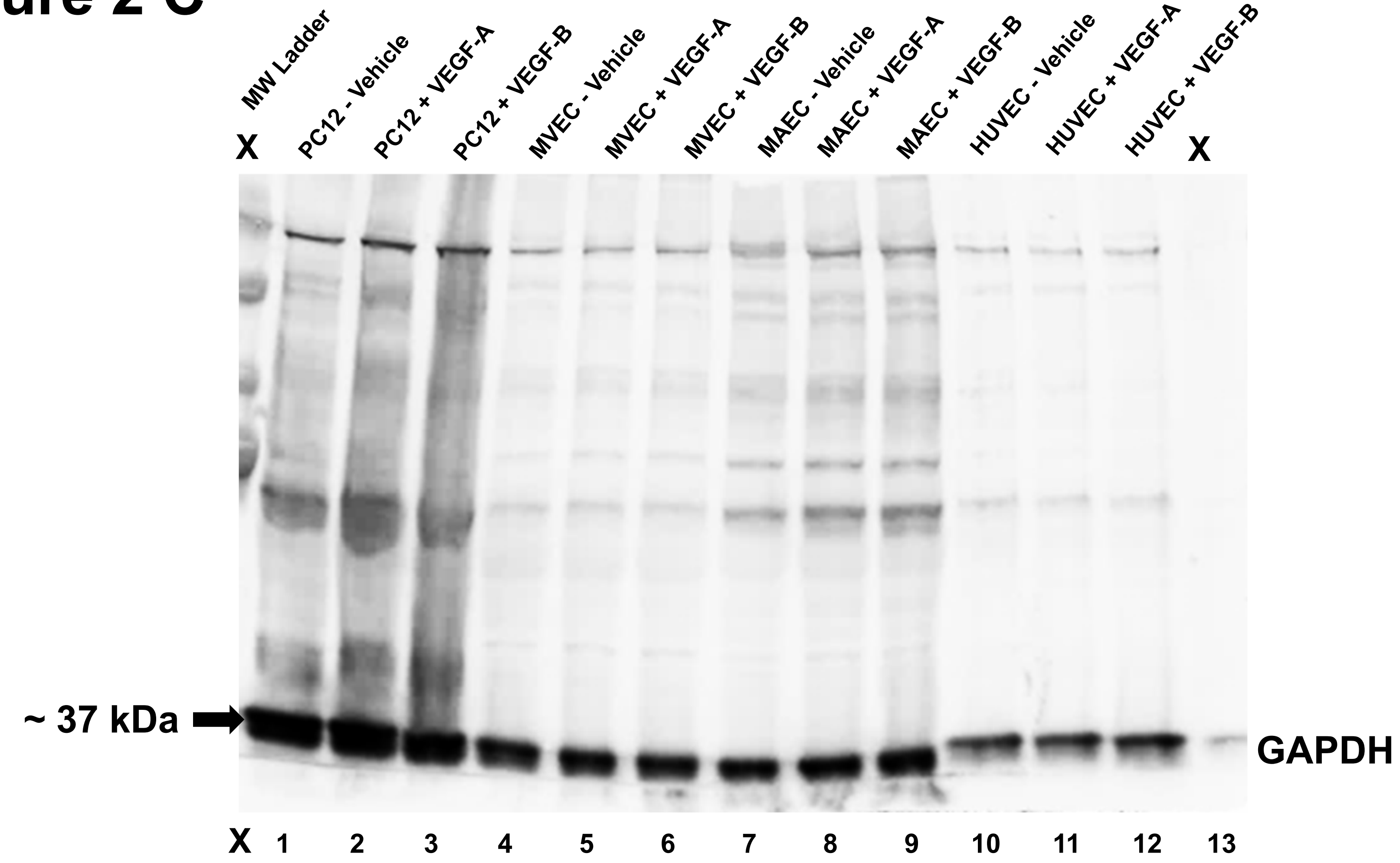

MW Ladder
PC12 - Vehicle
PC12 + VEGF-A
PC12 + VEGF-B
MVEC - Vehicle
MVEC + VEGF-A
MVEC + VEGF-B
MAEC - Vehicle
MAEC + VEGF-A
MAEC + VEGF-B
HUVEC - Vehicle
HUVEC + VEGF-A
HUVEC + VEGF-B
X
X
~ 37 kDa
GAPDH
X 1 2 3 4 5 6 7 8 9 10 11 12 13

Figure 3 A

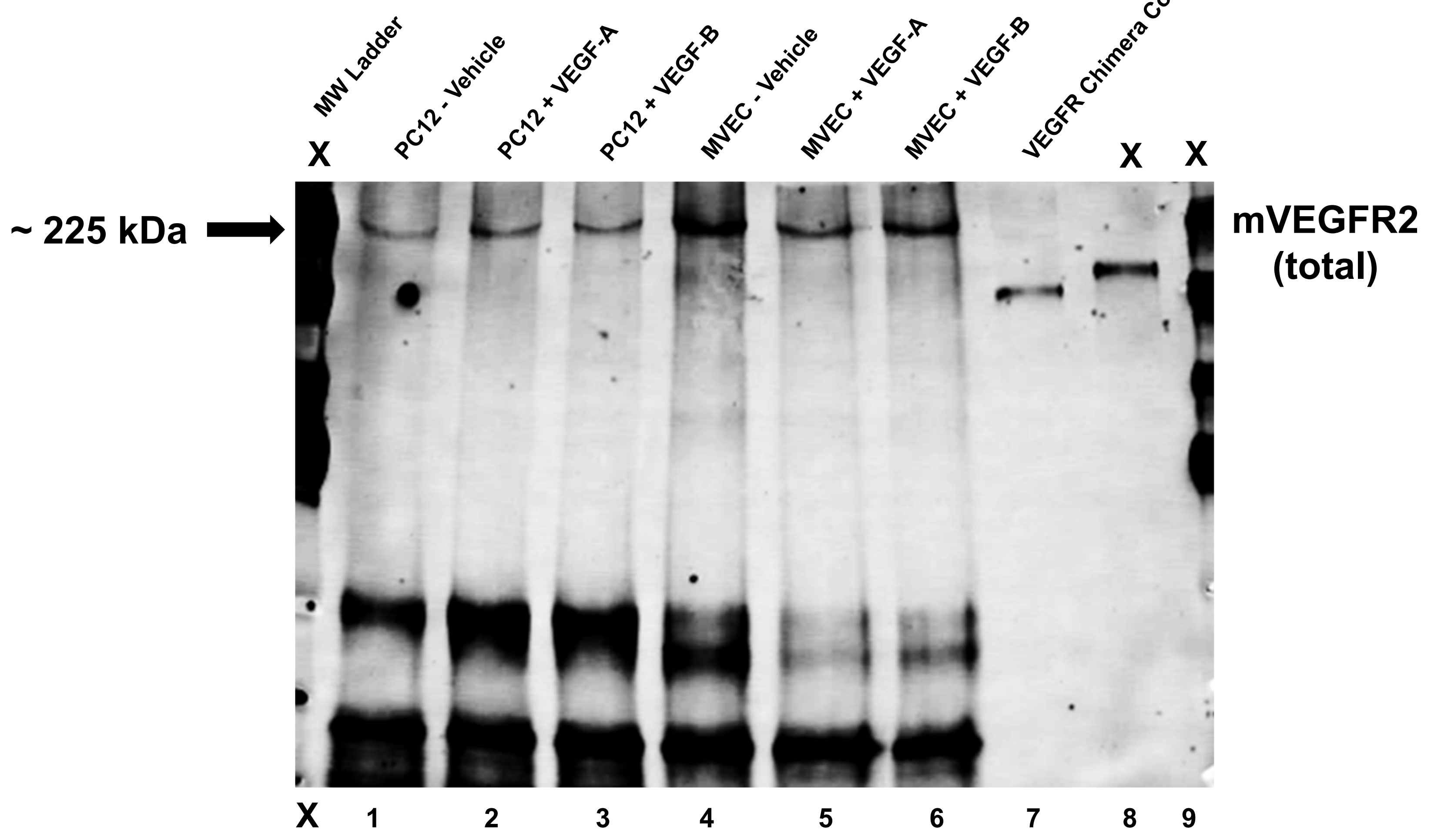
MW Ladder
PC12 - Vehicle
PC12 + VEGF-A
PC12 + VEGF-B
MVEC - Vehicle
MVEC + VEGF-A
MVEC + VEGF-B
VEGFR Chimera Control-1
X
X
X
~ 225 kDa
mVEGFR2
(total)
X
1
2
3
4
5
6
7
8
9

Figure 3 B

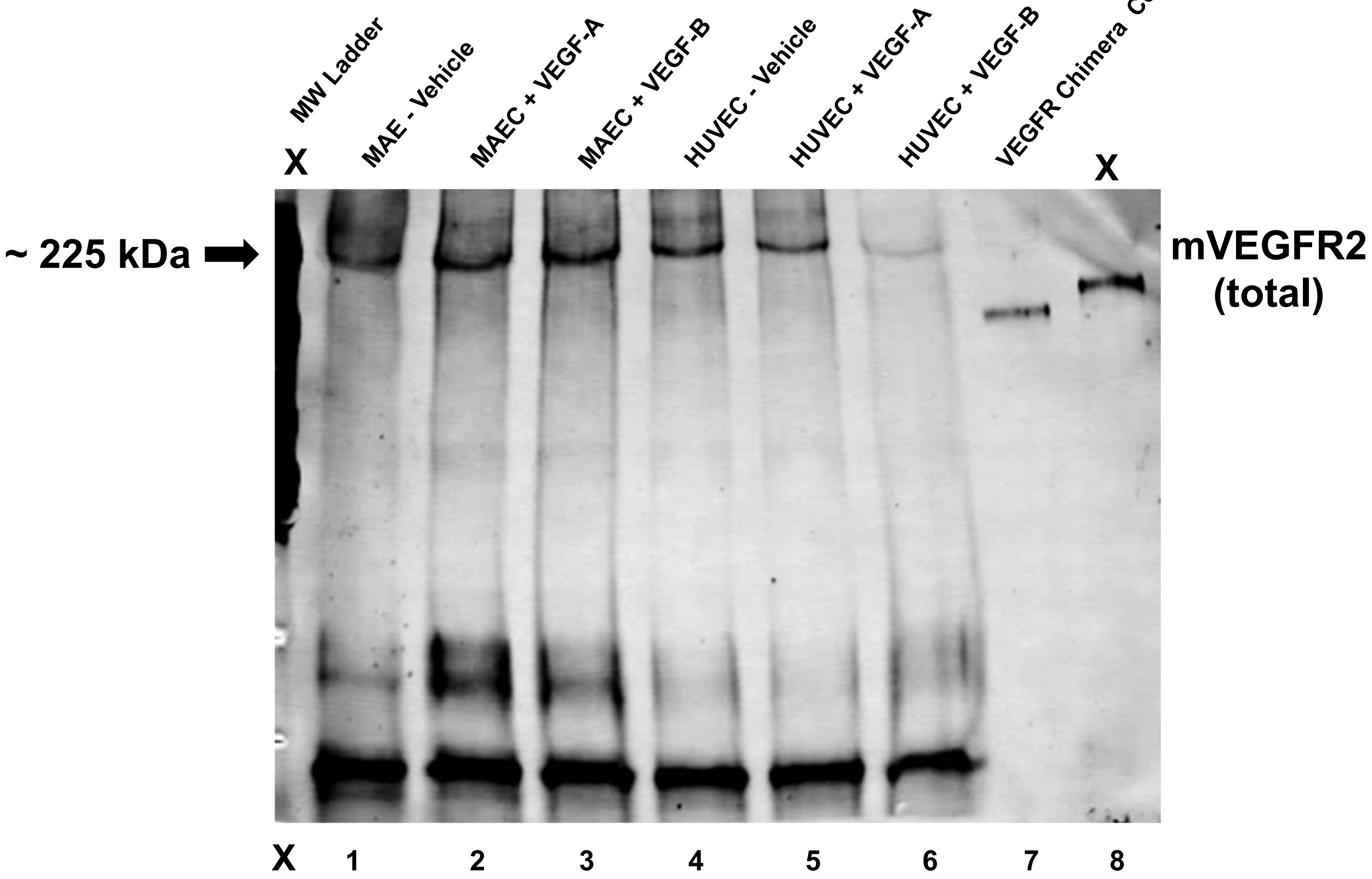
MW Ladder
MAE - Vehicle
MAEC + VEGF-A
MAEC + VEGF-B
HUVEC - Vehicle
HUVEC + VEGF-A
HUVEC + VEGF-B
VEGFR Chimera Control-1
X
X
~ 225 kDa
mVEGFR2
(total)
X
1
2
3
4
5
6
7
8

Figure 3 C

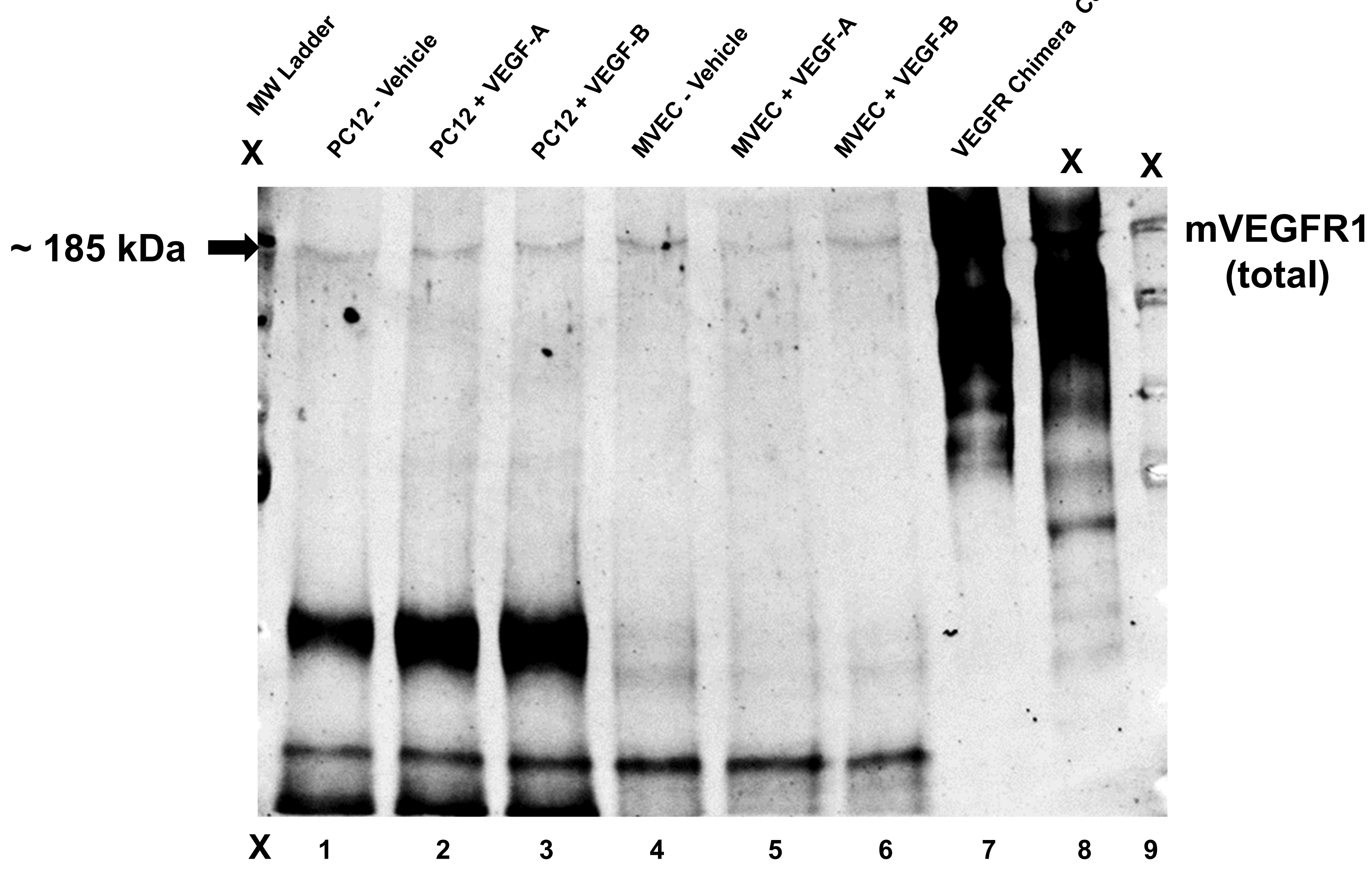
MW Ladder
PC12 - Vehicle
PC12 + VEGF-A
PC12 + VEGF-B
MVEC - Vehicle
MVEC + VEGF-A
MVEC + VEGF-B
VEGFR Chimera Control-1
X
X
X
~ 185 kDa
mVEGFR1
(total)
X
1
2
3
4
5
6
7
8
9

Figure 3 D

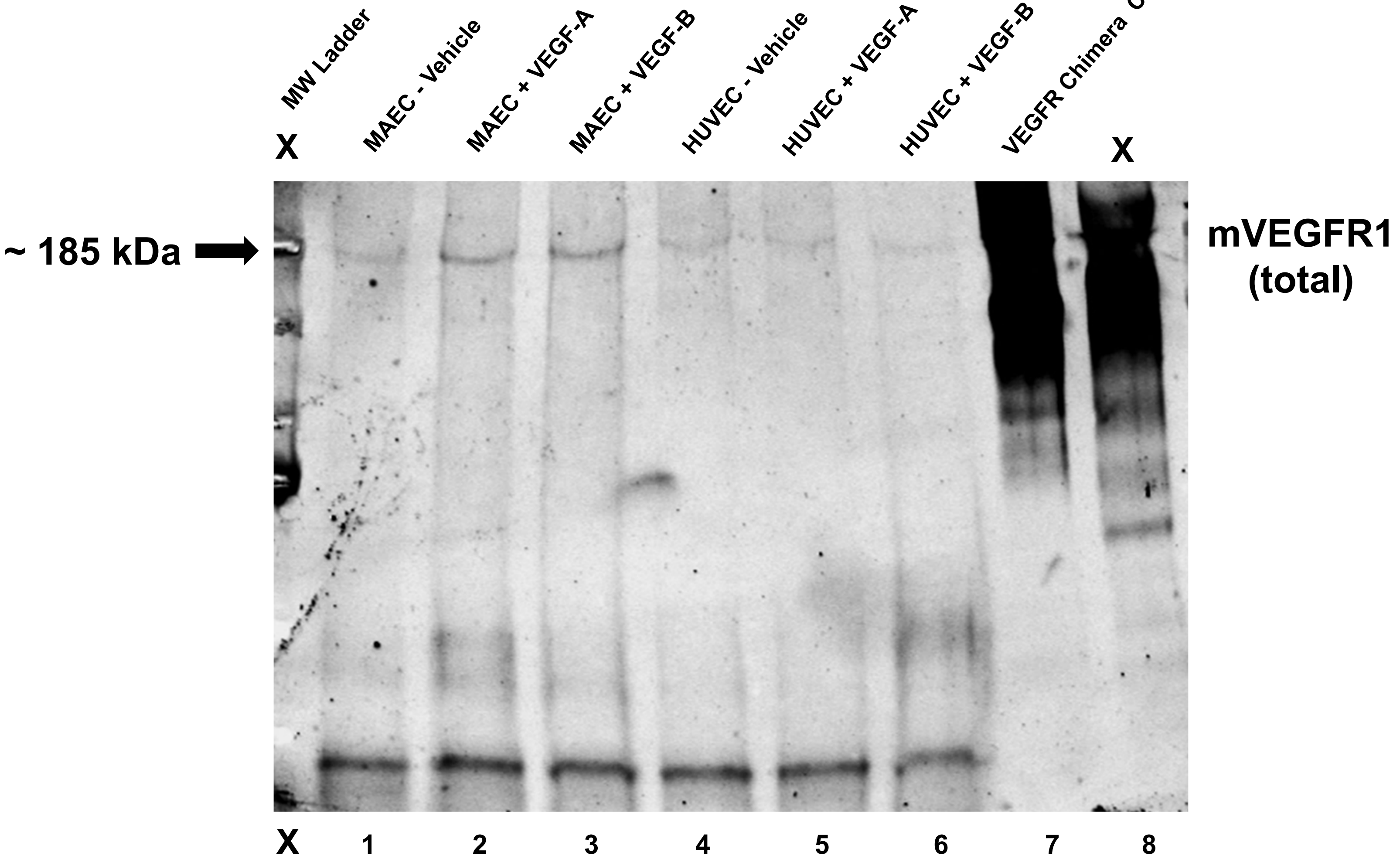
MW Ladder
MAEC - Vehicle
MAEC + VEGF-A
MAEC + VEGF-B
HUVEC - Vehicle
HUVEC + VEGF-A
HUVEC + VEGF-B
VEGFR Chimera Control-1
X
X
~ 185 kDa
mVEGFR1
(total)
X
1
2
3
4
5
6
7
8

Figure 3 E

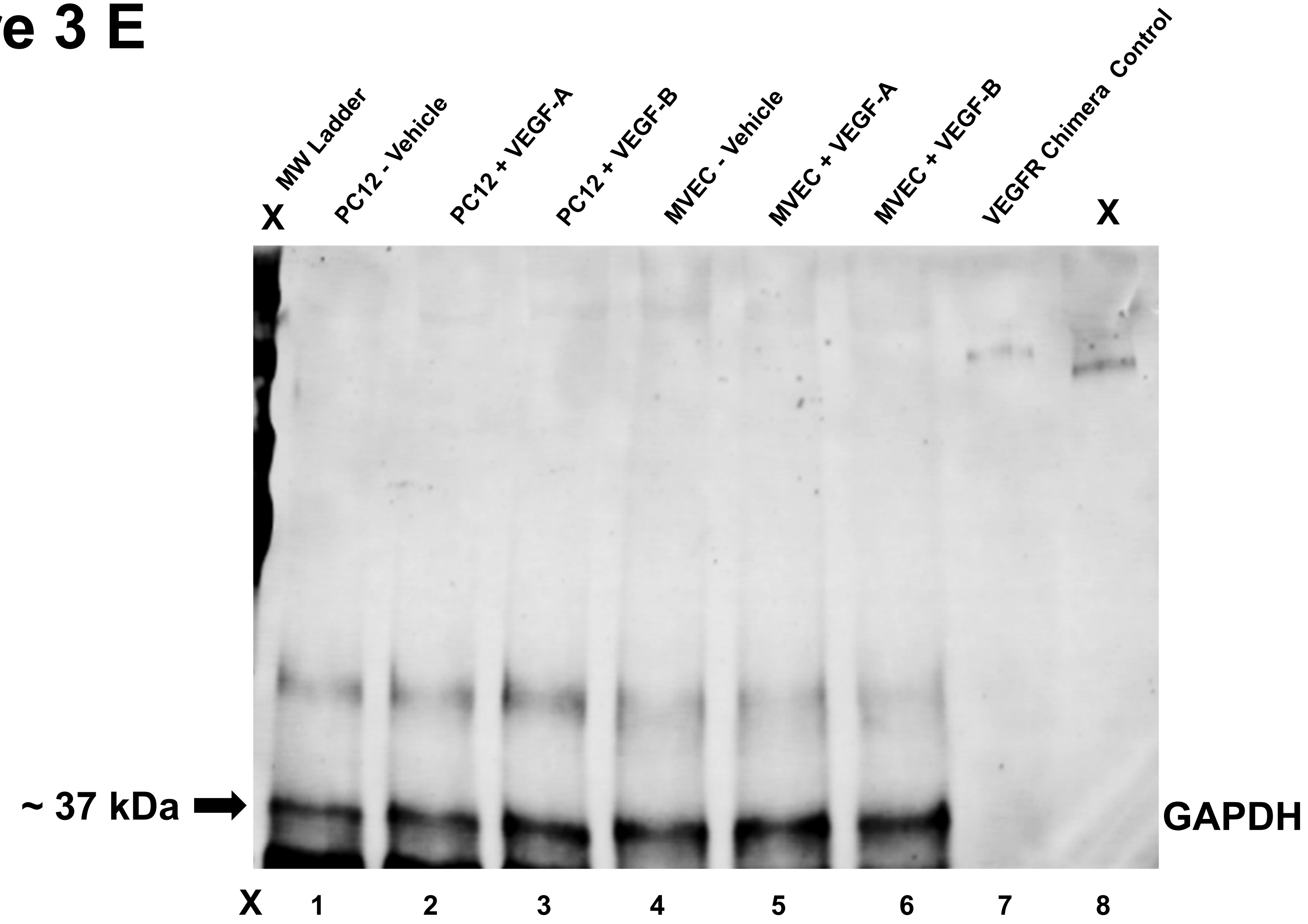
MW Ladder
PC12 - Vehicle
PC12 + VEGF-A
PC12 + VEGF-B
MVEC - Vehicle
MVEC + VEGF-A
MVEC + VEGF-B
VEGFR Chimera Control
X
X
~ 37 kDa
GAPDH
X
1
2
3
4
5
6
7
8

Figure 3 F

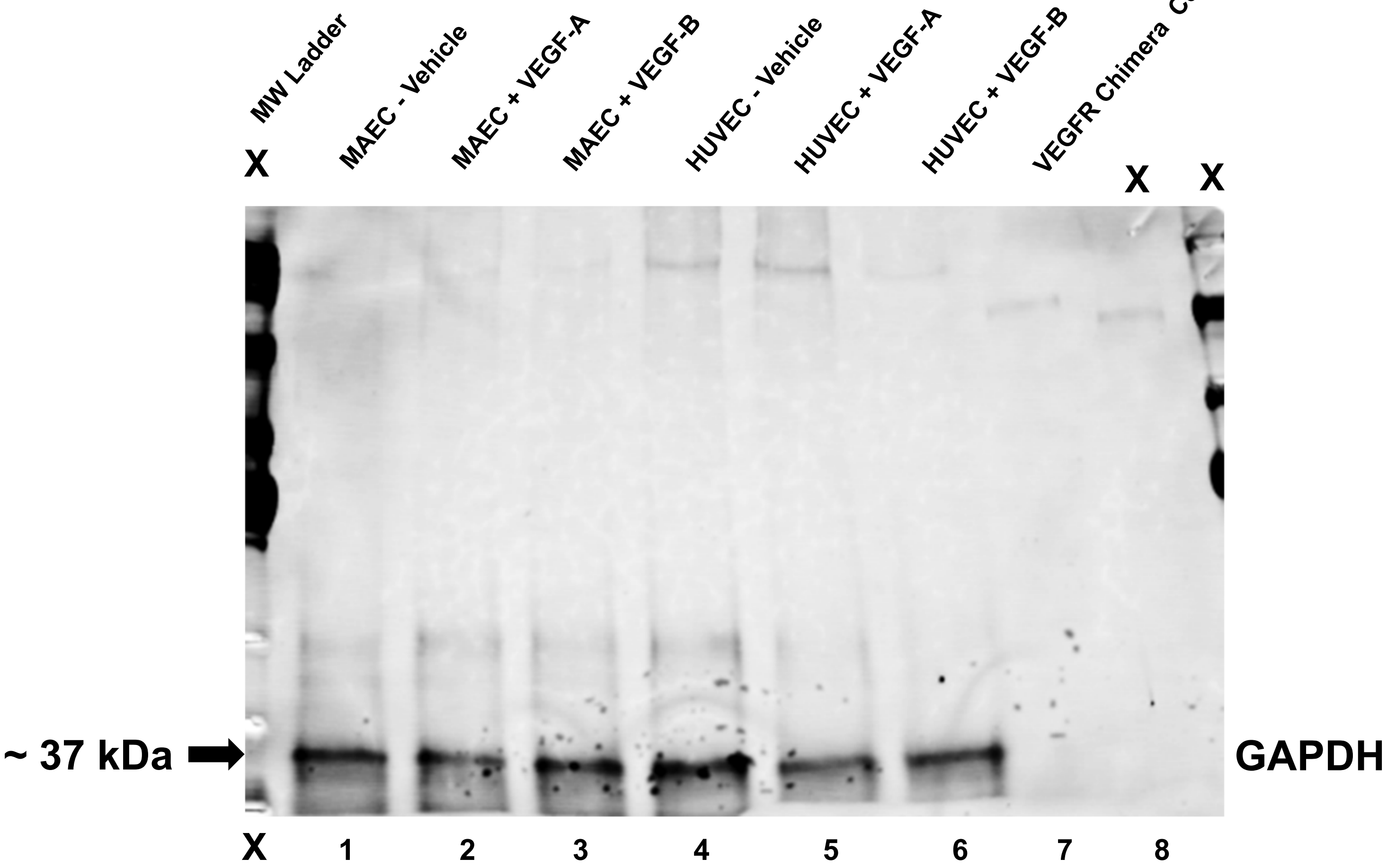

MW Ladder
MAEC - Vehicle
MAEC + VEGF-A
MAEC + VEGF-B
HUVEC - Vehicle
HUVEC + VEGF-A
HUVEC + VEGF-B
VEGFR Chimera Control-1
X
X
X
~ 37 kDa
GAPDH
X
1
2
3
4
5
6
7
8

Figure 3 G

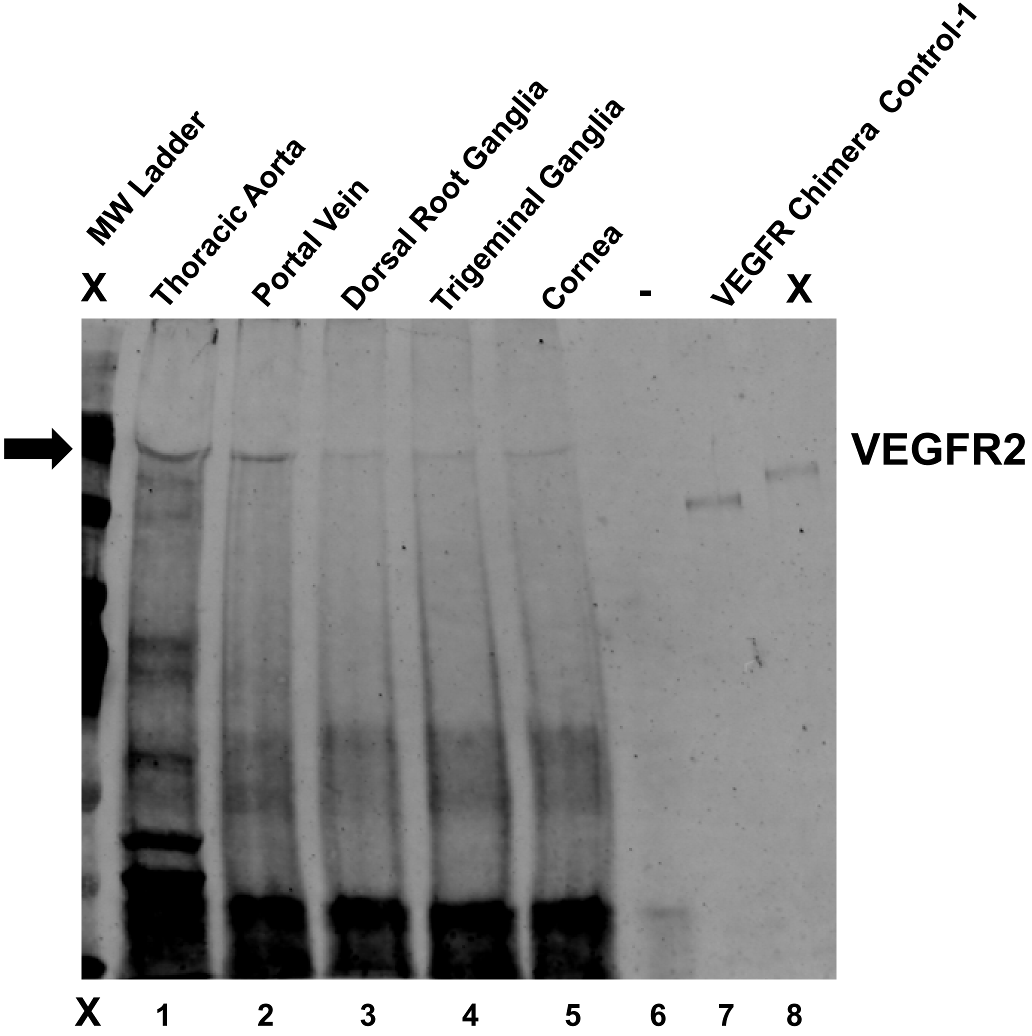

MW Ladder
Thoracic Aorta
Portal Vein
Dorsal Root Ganglia
Trigeminal Ganglia
Cornea
-
VEGFR Chimera
Control-1
X
X
VEGFR2
X
1
2
3
4
5
6
7
8

Figure 3 H

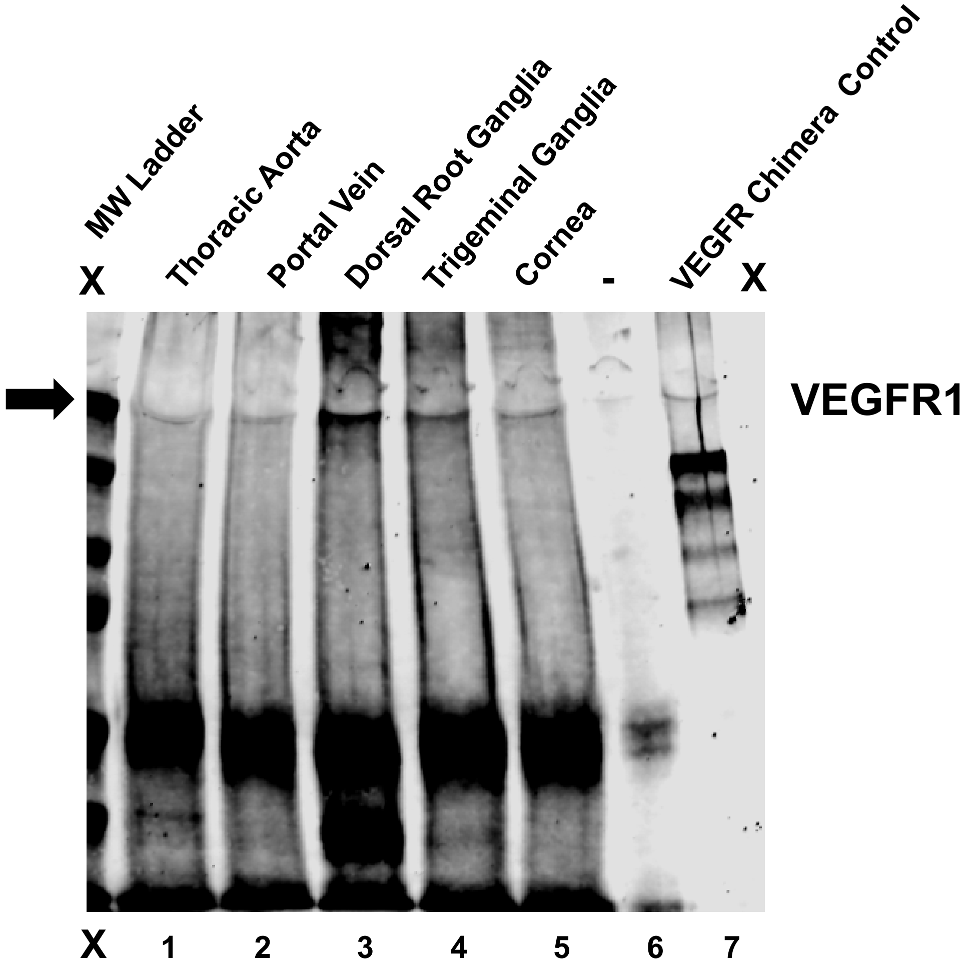
MW Ladder
Thoracic Aorta
Portal Vein
Dorsal Root Ganglia
Trigeminal Ganglia
Cornea
-
VEGFR Chimera Control
X
X
VEGFR1
X
1
2
3
4
5
6
7

Figure 3 I

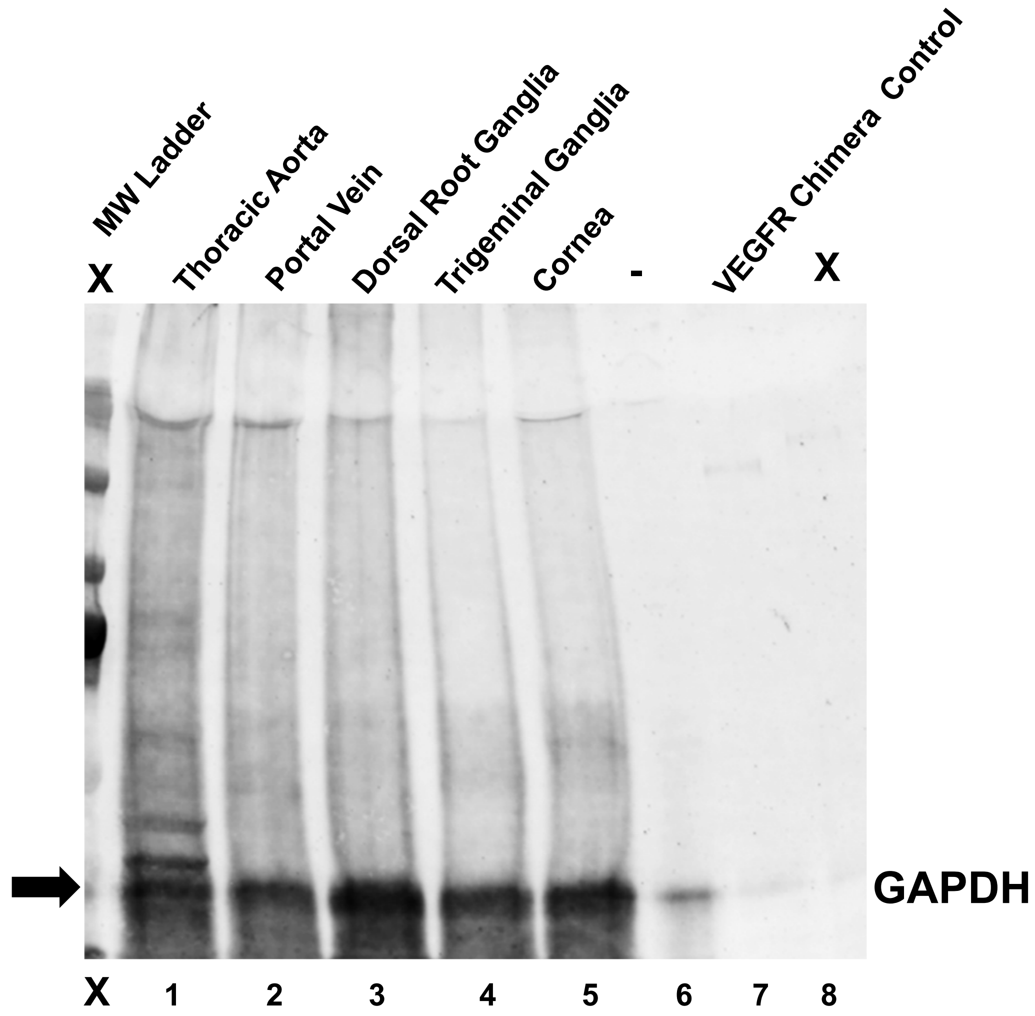
MW Ladder
Thoracic Aorta
Portal Vein
Dorsal Root Ganglia
Trigeminal Ganglia
Cornea
-
VEGFR Chimera Control
X
X
GAPDH
X 1 2 3 4 5 6 7 8
